# Different transcriptional responses of haploid and diploid *S. cerevisiae* strains to changes in cofactor preference of XR

**DOI:** 10.1186/s12934-020-01474-2

**Published:** 2020-11-13

**Authors:** Cai-Yun Xie, Bai-Xue Yang, Qing-Ran Song, Zi-Yuan Xia, Min Gou, Yue-Qin Tang

**Affiliations:** College of Architecture and Environment, Sichuan University, No. 24, South Section 1, First Ring Road, Chengdu, 610065 Sichuan China

**Keywords:** *Saccharomyces cerevisiae*, Xylose fermentation, XR-XDH pathway, CRISPR/Cas9, Transcriptomics, Bioethanol

## Abstract

**Background:**

Xylitol accumulation is a major barrier for efficient ethanol production through heterologous xylose reductase-xylitol dehydrogenase (XR-XDH) pathway in recombinant *Saccharomyces cerevisiae*. Mutated NADH-preferring XR is usually employed to alleviate xylitol accumulation. However, it remains unclear how mutated XR affects the metabolic network for xylose metabolism. In this study, haploid and diploid strains were employed to investigate the transcriptional responses to changes in cofactor preference of XR through RNA-seq analysis during xylose fermentation.

**Results:**

For the haploid strains, genes involved in xylose-assimilation (*XYL1*, *XYL2*, *XKS1*), glycolysis, and alcohol fermentation had higher transcript levels in response to mutated XR, which was consistent with the improved xylose consumption rate and ethanol yield. For the diploid strains, genes related to protein biosynthesis were upregulated while genes involved in glyoxylate shunt were downregulated in response to mutated XR, which might contribute to the improved yields of biomass and ethanol. When comparing the diploids with the haploids, genes involved in glycolysis and MAPK signaling pathway were significantly downregulated, while oxidative stress related transcription factors (TFs) were significantly upregulated, irrespective of the cofactor preference of XR.

**Conclusions:**

Our results not only revealed the differences in transcriptional responses of the diploid and haploid strains to mutated XR, but also provided underlying basis for better understanding the differences in xylose metabolism between the diploid and haploid strains.

## Background

Lignocellulosic biomass is regard as an abundant and sustainable feedstock for fuel ethanol production. Hydrolysis of lignocellulose primarily releases glucose and xylose. *Saccharomyces cerevisiae* as the traditional ethanol producer cannot utilize xylose. Heterologous xylose reductase-xylitol dehydrogenase (XR-XDH) pathway or xylose isomerase (XI) pathway is usually introduced into *S. cerevisiae* to enable xylose utilization [1]. Compared with XI strains, XR-XDH strains exhibit higher xylose consumption rate and ethanol productivity [2, 3]. However, xylitol accumulates seriously due to the cofactor imbalance between NADPH-preferring XR and NAD^+^-dependent XDH. Numerous efforts have been made to alter the cofactor preference of XR from NADPH to NADH, however, the decreased xylitol accumulation is usually accompanied by improved or reduced xylose utilization [4–7]. Several studies have discovered the interplays between the heterologous xylose pathway and the native host metabolism [8, 9], but how mutated XR affects the xylose metabolism remains unclear.

Xylose metabolism also depends on the choice of the host strains [9–11]. In comparison with laboratory strains, industrial strains are preferred for industrial application due to their excellent fermentabilities and better stress resistance [12–14]. Moreover, several studies have reported that ploidy changes have a notable effect on stress tolerance and ethanol productivity when fermenting glucose [15–17]. When fermenting xylose, haploids showed better performances than diploids [10]. Metabolomic and transcriptomic analyses have been carried out to compare diploid with haploid during glucose fermentation [18, 19], but the molecular basis for the distinct xylose fermentation capability of haploid and diploid strains remains obscure.

In our previous study, two haploid xylose-fermenting *S. cerevisiae* strains were constructed by expressing heterologous XR-XDH pathway using a haploid of industrial diploid strain KF7 [20] as host [21]. Strain HX57D expressing mutated XR had notably improved xylose consumption rate and ethanol yield compared with HX62W expressing native XR, which demonstrated that expressing mutated XR promoted xylose metabolism in the haploid strain [21]. In this study, two diploid xylose-fermenting strains were generated from KF7 by overexpressing XR (native or mutated) and XDH from *Scheffersomyces stipitis* as well as xylulokinase (XK) from *S. cerevisiae*. A CRISPR/Cas9-mediated method was adopted to enable rapid and maker-less integration of genes into the diploid *S. cerevisiae* strain. Although the diploid expressing mutated XR (strain A) had a much higher ethanol yield than the diploid expressing wild XR (strain B), the xylose consumption rates of both strains were similar. The effect of mutated XR on xylose metabolism was found to be closely correlated with the ploidy of strains. Comparative transcriptome analysis was performed to unravel the global transcriptional responses of the haploid and diploid strains (with the same genetic background) to mutated XR when fermenting xylose. The research provided theoretical guidance for the construction of efficient xylose-fermenting strains.

## Results and discussion

### Effect of mutated XR and increased ploidy on xylose fermentation

**Fig. 1:**
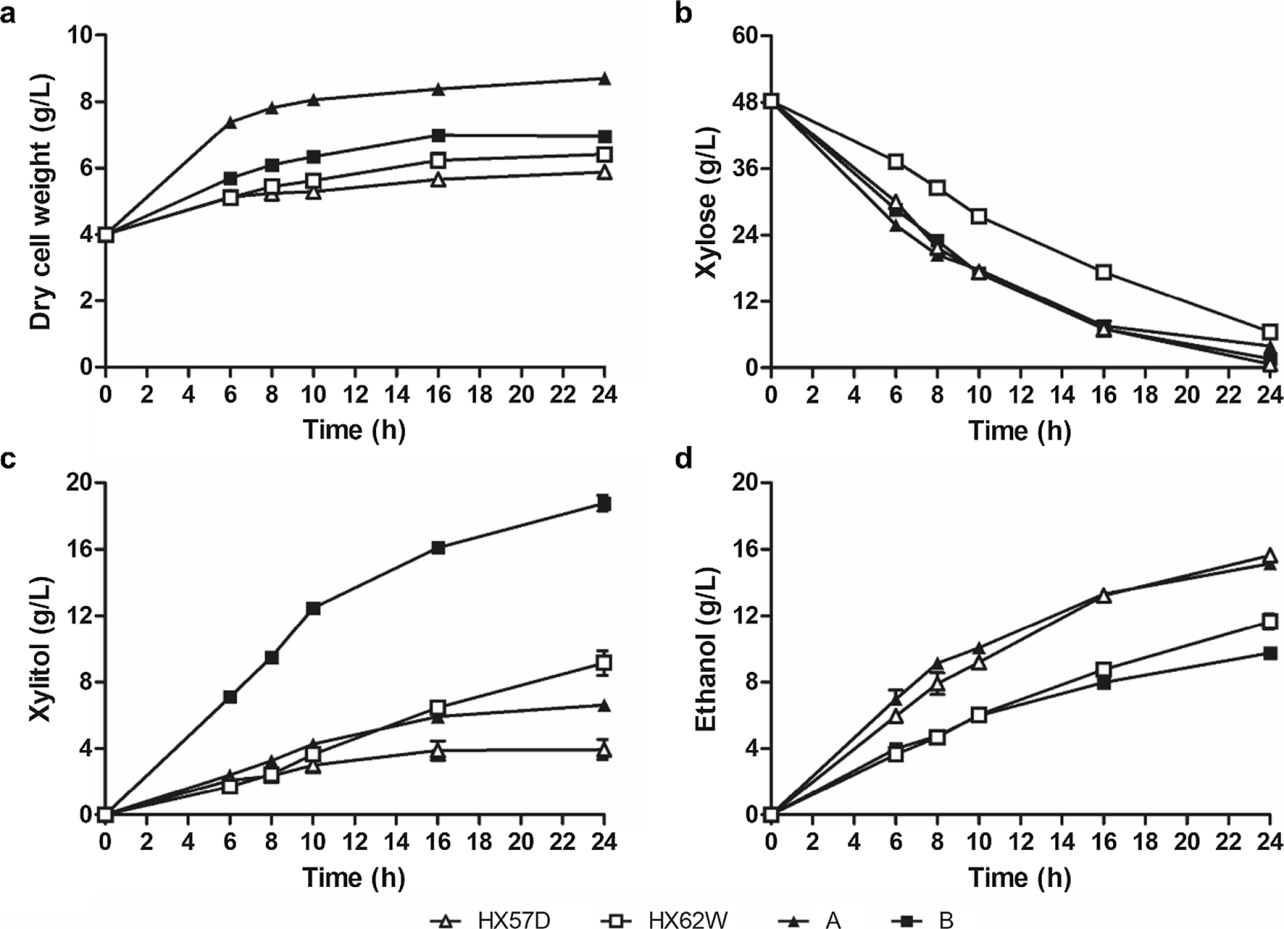
Batch fermentation of HX57D (open triangles), HX62W (open squares), A (closed triangles), and B (closed squares) using YPX50 medium. **a** Dry cell weight, **b** Xylose, **c** Xylitol, **d** Ethanol. The initial inoculum was 4 g /L DCW. The average values and standard derivations (error bars) for three independent experiments are presented

Two diploid xylose-fermenting strains A and B were constructed. Diploid A and haploid HX57D expressed double sites-mutated XR (K270R/N272D), while diploid B and haploid HX62W expressed native XR. The performance of these strains was compared when fermenting YPX50 (Fig. 1, Table 1).

**Table 1:** Fermentation characteristics of strains using YPX50 with an initial inoculum of 4 g/L DCW

	Q ^a^ (g/L/h)		Y ^b^ (g/g consumed xylose)			Enzymatic activity (U/mg) ^d^			[NADPH]/[NADH] ^e^
	Xylose	Ethanol	Xylitol	Ethanol	Biomass ^c^	NADPH-XR	NADH-XR	XDH	
HX57D	2.58 ± 0.01	0.83 ± 0.01	0.09 ± 0.01	0.32 ± 0.00	0.04 ± 0.00	0.65 ± 0.03	0.86 ± 0.01	11.24 ± 0.72	0.76
HX62W	1.93 ± 0.01	0.55 ± 0.01	0.21 ± 0.01	0.28 ± 0.01	0.07 ± 0.00	0.52 ± 0.03	0.36 ± 0.02	6.61 ± 0.09	1.45
A	2.54 ± 0.04	0.83 ± 0.00	0.15 ± 0.00	0.33 ± 0.00	0.11 ± 0.00	0.10 ± 0.00	0.31 ± 0.01	2.30 ± 0.00	0.31
B	2.58 ± 0.07	0.50 ± 0.01	0.39 ± 0.01	0.19 ± 0.00	0.07 ± 0.00	0.44 ± 0.01	0.26 ± 0.00	3.83 ± 0.14	1.67

Data for enzymatic activity were measured at 8 h of fermentation. Other data were calculated by 16 h fermentation. Data are means ± SD from three independent experiments

^a^Xylose consumption rate or ethanol production rate (g/L/h)

^b^Yield of xylitol and ethanol (g/g consumed xylose)

^c^Yield of biomass (g DCW/g consumed xylose)

^d^Specific activity (U/mg protein)

^e^Specific activity with NADPH/specific activity with NADH

After 16 h of fermentation, the xylose consumption rate of HX57D was 34% higher than that of HX62W, while the consumption rate of strain A did not increase compared with strain B (Table 1). The ethanol yields of HX57D and A were higher than those of HX62W and B, respectively, whereas the xylitol yields were in reverse (Table 1). Moreover, HX57D had 43% lower biomass yield than HX62W, whereas A had 57% higher biomass yield than B (Table 1). The specific activities of XR and XDH measured at 8 h of fermentation were summarized in Table 1. The NADPH/NADH ratios (represented for the ratio of NADPH- and NADH-dependent XR activities) of HX62W and B were greater than 1, while those of HX57D and A were less than 1, which demonstrated that the cofactor preference of XR(K270R/N272D) was altered from NADPH to NADH. Moreover, the NADPH-, NADH-dependent XR, and XDH activities of HX57D were 25%, 139%, and 70%, respectively, higher than those of HX62W (Table 1). The higher activities of both XR and XDH in the haploid strains might contribute to the improved xylose utilization, which was consistent with previous studies [22, 23]. When comparing diploid A with B, the NADH-dependent XR activity was 19.2% higher but the NADPH-dependent XR and XDH activities were reduced by 77.3% and 39.9%, respectively (Table 1). The sharply decreased NADPH-dependent XR activity might result in decreased XDH activity and further limited xylose utilization. On the other hand, the increased NADPH-dependent XR activity in HX57D implied an increased demand for NADPH from XR, which might result in altered NADPH generation and further affect biomass formation [24]. The increased biomass yield of strain A was also correlated with the decreased NADPH-dependent XR activity. In summary, the haploid and diploid strains had different xylose metabolic responses to mutated XR.

To investigate the differences in xylose metabolism between diploids and haploids, strains A and B were compared with HX57D and HX62W, respectively. XR-mutated strain A had similar xylose consumption rate and ethanol yield but higher biomass yield compared with HX57D (Table 1). However, HX57D accumulated slightly more glycerol than A (data not shown). XR-native strain B had higher xylose consumption rate but lower ethanol yield than HX62W (Table 1). The diploid strains had higher xylitol yields than the haploid strains, irrespective of the cofactor preference of XR (Table 1). Moreover, the XR and XDH activities of the diploids were also much lower than that of the haploids (Table 1). Our results supported the previous discovery that increasing ploidy did not improve xylose fermentation [10]. There was no significant correlation between biomass formation and ploidy when fermenting xylose in the present study. This result conflicted with the previous finding that biomass formation decreased with the increase of ploidy when grew on glucose [16].

### Transcriptional differences between haploid and diploid cells in response to mutated XR

The global transcriptional responses of the haploid and diploid strains to changes in cofactor preference of XR were analyzed by using RNA-seq. Among the 6448 genes of the yeast genome, only differentially expressed genes (DEGs) (false discovery rate (FDR) < 0.05 and |log_2_fold change (FC)|≥ 1) were further analyzed. To validate the expression profiles obtained by RNA-seq analysis, the expressions of the three genes involved in xylose-utilizing pathway (*XYL1*, *XYL2*, *XKS1*) and two genes involved in glycolysis (*ENO1*, *ENO2*) which showed different expression levels among strains were analyzed by quantitative reverse-transcription PCR (qRT-PCR). The results of qRT-PCR were highly consistent with those of the transcriptome analysis (Additional file 1: Fig. S1), indicating the validity of transcriptome data.

**Fig. 2:**
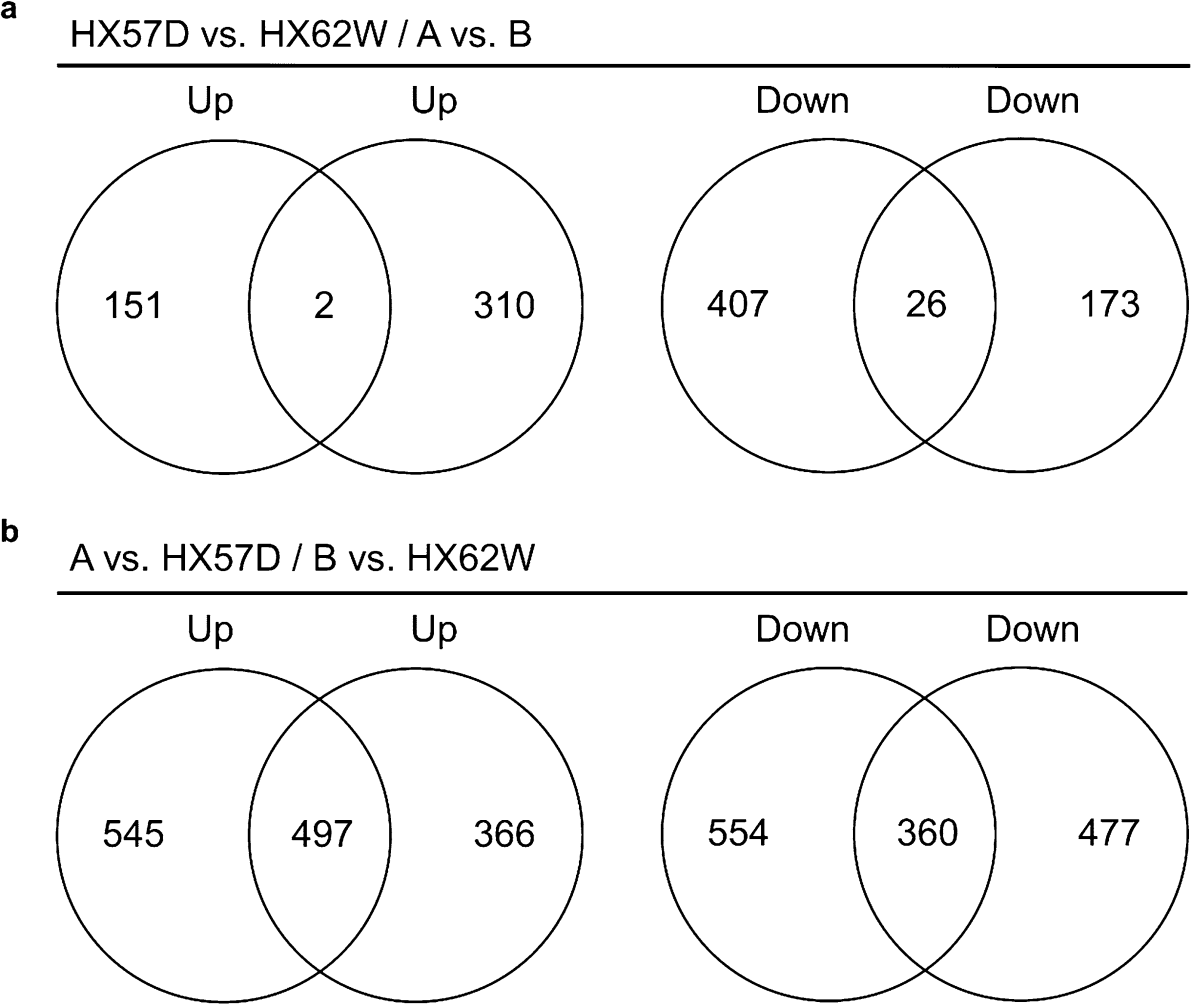
Venn diagram of DEGs in (**a**) XR-mutated strains compared with wild strains, and (**b**) the diploids compared with the haploids. Samples were taken at 8 h of xylose fermentation. Averages of biological triplicates are presented

The transcriptome profile was organized into two relevant pairwise comparisons: HX57D vs. HX62W and A vs. B. A total of 586 and 511 DEGs were found in the comparison of HX57D vs. HX62W and A vs. B, respectively (Additional file 1: Fig. S2a, b). However, only 28 DEGs were shared by the both comparisons (Fig. 2a), which implied that the haploid and diploid strains had distinct responses to mutated XR.

### KEGG pathway analysis

**Fig. 3:**
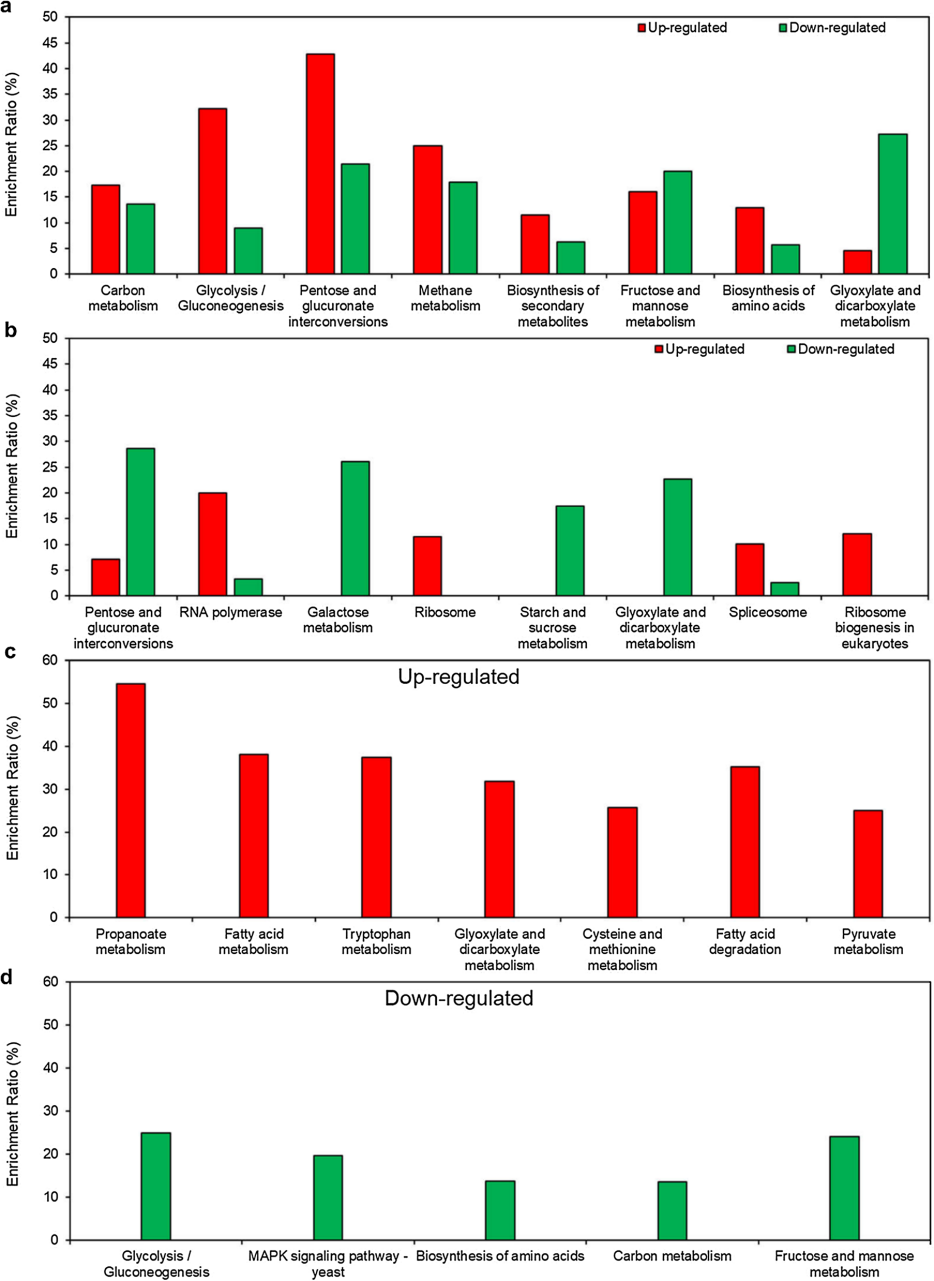
Enriched KEGG pathways for DEGs between (**a**) HX57D and HX62W (p < 0.02), (**b**) A and B (p < 0.04), and for significantly (**c**) upregulated and (**d**) downregulated genes between the diploids and the haploids (p < 0.01). The enrichment ratio of each KEGG pathway was the number of DEGs involved in each KEGG pathway to the number of total genes in each pathway

Based on the KEGG enrichment analysis, eight pathways were significantly enriched for DEGs between HX57D and HX62W (p < 0.02) (Fig. 3a, Additional file 1: Table S1). These pathways were largely involved in carbohydrate metabolism, including carbon metabolism, glycolysis/gluconeogenesis, pentose and glucuronate interconversions, fructose and mannose metabolism as well as glyoxylate and dicarboxylate metabolism (Fig. 3a). Similarly, four KEGG pathways (pentose and glucuronate interconversions, galactose metabolism, starch and sucrose metabolism, glyoxylate and dicarboxylate metabolism) related to carbohydrate metabolism were also enriched in the comparison group A vs. B (p < 0.04) (Fig. 3b, Additional file 1: Table S1). These results indicated that carbohydrate metabolism was notably affected by mutated XR in both the haploid and diploid strains. Further discussion was expanded later.

On the other hand, four pathways associated with protein biosynthesis, i.e., RNA polymerase, ribosome, spliceosome, and ribosome biogenesis in eukaryotes, were uniquely enriched in group A vs. B, and most DEGs in these pathways were upregulated in strain A compared with strain B (Fig. 3b). It has been reported that the enhanced expression of genes involved in protein biosynthesis was positively correlated with the increased growth rate of cells [25, 26]. For strain A, the improved protein biosynthesis was consistent with the higher biomass yield compared with strain B. Moreover, enhanced ribosome synthesis has been proved to be beneficial to xylose utilization [27].

### Sugar transporters

Xylose uptake depends on native hexose transporters in *S. cerevisiae*, which is composed of 18 genes from the *HXT*s family and galactose permease *GAL2* [28]. When comparing HX57D with HX62W, the transcript levels of *HXT4*, *HXT5*, *HXT10*, *HXT13*, *HXT17*, and *GAL2* were significantly changed (Table 2). Transporters with extremely low absolute transcript abundances were not discussed in this study. The main xylose transporter *HXT4* [29] was induced in response to mutated XR in the haploid strain, whereas the non-fermentation carbon source-inducible transporter *HXT5* [30] was repressed. For the diploid strains, *HXT2* and *HXT15* were significantly downregulated in response to mutated XR (Table 2). *HXT2*, a high-affinity permease, allowed xylose consumption with the same rate as glucose [31]. It seemed that the expression of different hexose transporters might be affected by the contents of NADH, NADPH, and their ratio inside cells as well as the xylose consumption rate. However, the correlation of the expression of transporters with these factors needs further investigation.

**Table 2:** The expression level of genes encoding hexose transporters in different strains

Gene	FPKM ^a^				Log_2_FC ^b^			
	HX57D	HX62W	A	B	HX57D vs. HX62W	A vs. B	A vs. HX57D	B vs. HX62W
*HXT1*	9.6 ± 0.9	14.0 ± 0.9	5.5 ± 0.4	3.5 ± 0.4	N	0.6	− 1.0	− 1.9
*HXT2*	30.2 ± 9.2	59.7 ± 5.4	57.2 ± 6.8	201.2 ± 29.5	− 0.8	− 1.8	0.8	1.9
*HXT3*	57.3 ± 10.8	58.8 ± 4.4	13.1 ± 1.8	11.1 ± 1.1	N	N	− 2.3	− 2.3
*HXT4*	471.8 ± 62.2	123.3 ± 10.6	39.4 ± 6.2	27.9 ± 0.9	2.2	0.5	− 3.8	− 2.0
*HXT5*	76.6 ± 23.3	206.3 ± 22.8	213.4 ± 23.0	296.6 ± 5.2	− 1.2	− 0.5	1.3	0.6
*HXT6*	275.4 ± 29.5	251.6 ± 30.2	108.0 ± 20.9	130.1 ± 31.0	N	N	− 1.5	− 0.8
*HXT7*	583.7 ± 41.7	548.4 ± 87.6	244.6 ± 40.7	264.8 ± 51.6	N	N	− 1.4	− 1.0
*HXT8*	16.2 ± 3.1	16.3 ± 2.0	17.7 ± 1.4	15.1 ± 1.2	N	N	N	N
*HXT9*	6.1 ± 0.7	4.6 ± 0.4	5.7 ± 0.1	7.9 ± 0.6	0.6	− 0.5	N	0.9
*HXT10*	1.0 ± 0.3	2.6 ± 0.5	3.1 ± 0.4	5.6 ± 0.5	− 1.1	− 0.9	1.4	1.2
*HXT11*	15.2 ± 0.7	15.5 ± 1.1	13.7 ± 0.6	18.0 ± 1.1	N	− 0.4	N	N
*HXT12*	5.9 ± 1.1	5.0 ± 1.0	5.4 ± 0.5	7.3 ± 0.1	N	− 0.4	N	0.6
*HXT13*	3.1 ± 0.7	9.3 ± 0.6	223.3 ± 7.5	261.1 ± 17.0	− 1.4	− 0.2	6.0	4.9
*HXT14*	1.5 ± 0.6	1.9 ± 0.4	1.6 ± 0.4	1.6 ± 0.4	N	N	N	N
*HXT15*	3.7 ± 0.6	6.4 ± 0.8	19.7 ± 1.5	39.2 ± 4.9	N	− 1.0	2.2	2.7
*HXT16*	1.8 ± 0.3	3.0 ± 0.4	7.4 ± 0.8	12.0 ± 1.4	N	− 0.7	1.9	2.1
*HXT17*	1.5 ± 0.4	5.5 ± 0.5	123.1 ± 4.1	172.7 ± 9.3	− 1.7	− 0.5	6.2	5.1
*GAL2*	8.1 ± 1.4	18.4 ± 0.7	57.9 ± 1.9	82.9 ± 4.9	− 1.0	− 0.5	2.7	2.3

^a^Values are given as the average of three biological duplicates ± standard deviation

^b^Values are given as the average of three biological duplicates

### Central carbon metabolism

**Fig. 4:**
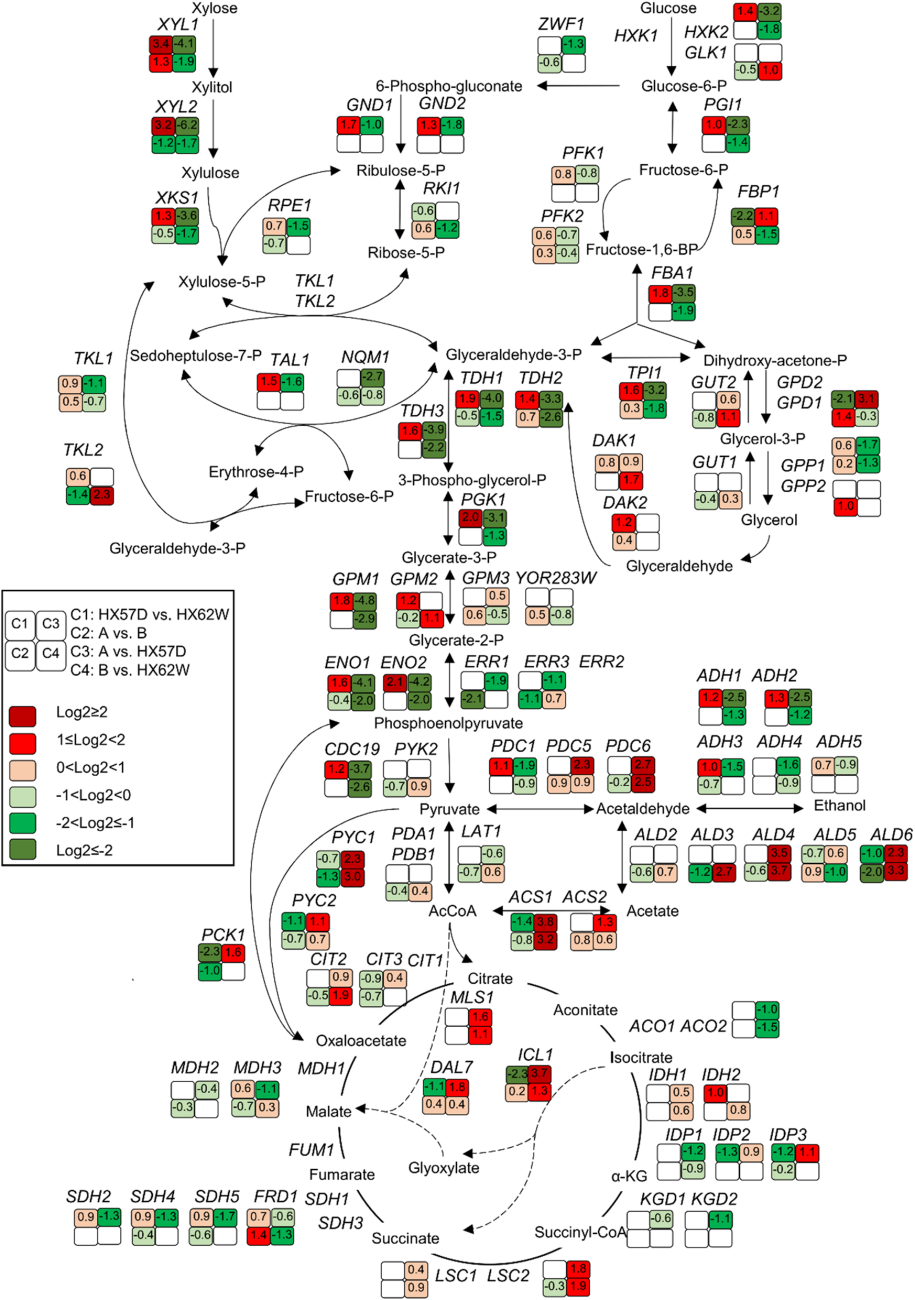
Transcriptional profiling of DEGs involved in the central carbon metabolism during xylose fermentation. C1, C2, C3, and C4 designate pairwise comparisons of HX57D vs. HX62W, A vs. B, A vs. HX57D, and B vs. HX62W respectively. The value of log_2_FC is presented as the average of biological triplicates. Varied colors represent for different change levels

Xylose is converted to ethanol through the heterologous xylose assimilating pathway, pentose phosphate pathway (PPP), glycolysis pathway and ethanol fermentation pathway [13]. To reveal the effect of mutated XR on xylose metabolism in the haploid and diploid strains, expression of genes involved in central carbon metabolism was investigated (Fig. 4).

When comparing HX57D with HX62W, genes in the upper xylose assimilating pathway (*XYL1*, *XYL2, XKS1*) were upregulated, which implied an enhanced xylose flux into central carbon metabolism. *TAL1*, encoding the major transaldolase in the non-oxidative PPP, was also upregulated in HX57D. Our previous study reported that overexpression of *TAL1* improved xylose fermentation as well as inhibitor tolerance [32]. In the oxidative PPP, *GND1* and *GND2*, encoding 6-phosphogluconate dehydrogenase, were significantly upregulated, which suggested that more NADPH was available for the xylose reduction in HX57D. A previous study observed that the xylose flux through glycolysis was limited by low PPP activity [33]. In this study, the higher expression levels of *TAL1*, *GND1*, and *GND2* might contribute to the efficient xylose utilization of HX57D.

Most glycolysis genes were significantly upregulated in HX57D (Fig. 4). An active glycolysis could benefit the xylose utilization [11, 34]. Moreover, genes involved in the alcohol fermentation pathways (*PDC1*, *ADH1*, *ADH2*, and *ADH3*) were also upregulated in HX57D. *PDC1* and *ADH1*, encoding the major pyruvate decarboxylase and alcohol dehydrogenase, respectively, are vital for ethanol fermentation in *S. cerevisiae*. *ADH3*, encoding mitochondrial alcohol dehydrogenase, was found to involve in a redox shuttle in *S. cerevisiae* [35].

The expression levels of *ALD6* (encoding aldehyde dehydrogenase) and *ACS1* (encoding acetyl-coenzyme A synthetase) decreased significantly in HX57D than in HX62W, suggesting that acetaldehyde was utilized for ethanol production instead of growth during xylose fermentation. Moreover, genes involved in the tricarboxylic acid (TCA) cycle (*IDP2* and *IDP3*), glyoxylate shunt (*ICL1* and *DAL7*), and gluconeogenesis (*FBP1*, *PYC2*, and *PCK1*) were downregulated in HX57D. Taken together, xylose was more likely to be sensed as a fermentable carbon source by HX57D compared with HX62W. These results confirmed the previous finding that reducing the carbon fluxes in futile cycle such as gluconeogenesis, TCA cycle and glyoxylate shunt was crucial to achieve the optimal ethanol production from xylose [8, 36].

**Fig. 5:**
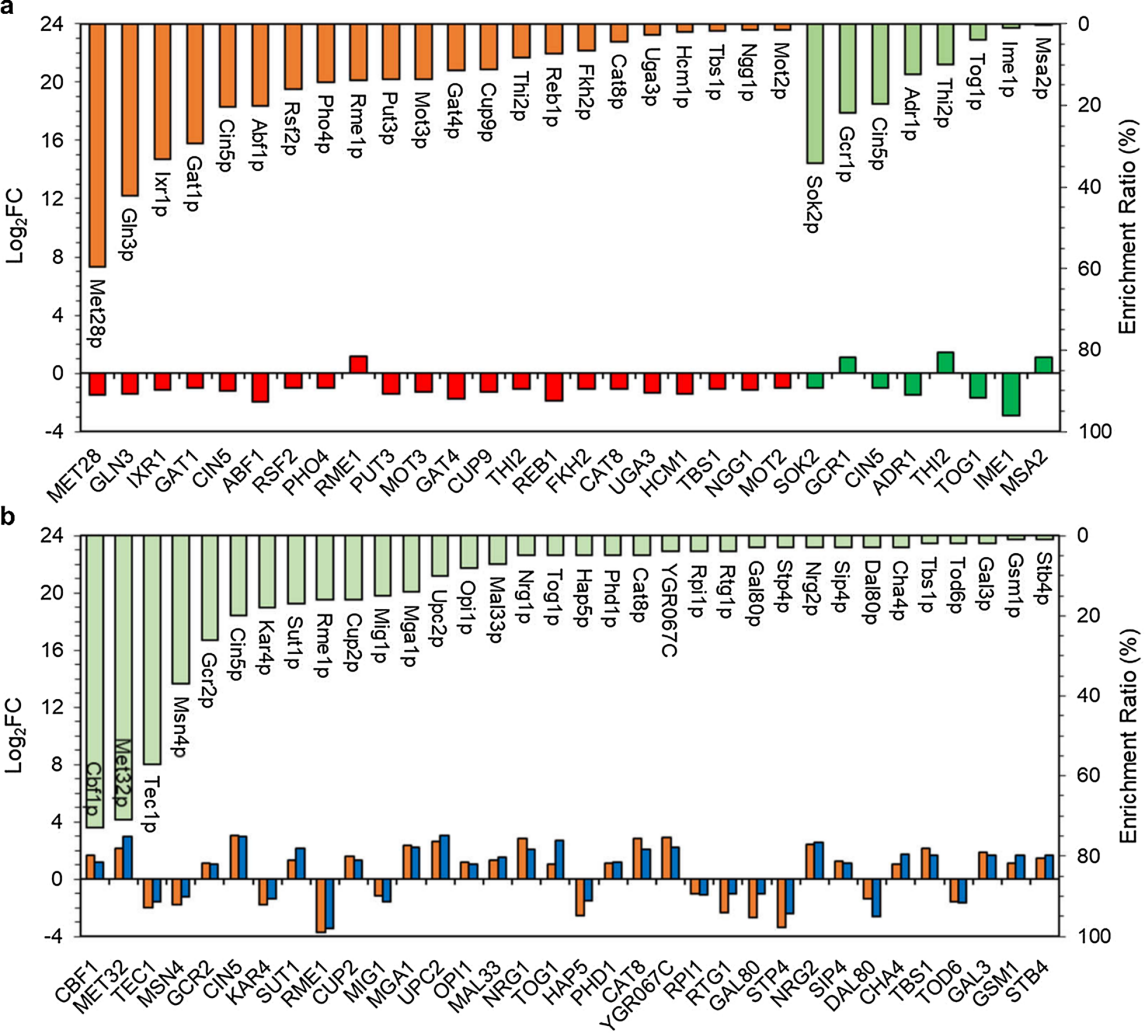
TFs with significantly different expression levels in groups (**a**) HX57D vs. HX62W (red) and A vs. B (green), respectively; and (**b**) both groups A vs. HX57D (orange) and B vs. HX62W (blue). The enrichment ratio of each TF was the number of DEGs regulated by the TF to the number of total DEGs

In the glycerol catabolism pathway, the expression level of the key glycerol-producing gene *GPD1* (encoding NADH-dependent glycerol-3-phosphate dehydrogenase) decreased in HX57D, while that of glycerol-utilizing gene *DAK2* (encoding dihydroxyacetone kinase) increased. The results indicated a decreased glycerol formation induced by mutated XR in the haploid strain. The accumulation of glycerol was an effective route for redox balancing in *S. cerevisiae*, which can re-oxidize the excess NADH generated from biomass formation [37]. HX57D might have a lower demand for NADH reoxidation via glycerol formation due to its lower biomass yield than HX62W. However, strain HX57D produced slightly more glycerol than HX62W after 24 h of fermentation (data not shown). It was speculated that the strain HX62W might retain most glycerol intracellularly for its role in osmo-tolerance [38]. The hypothesis was supported by the facts that the osmotic stress-related transcription factors (TFs), Cin5p and Mot3p, had higher expression levels in HX62W than in HX57D (Fig. 5a).

By contrast, few genes involved in central carbon metabolism notably changed in response to mutated XR in the diploid strain (Fig. 4). The expression level of *XYL1* increased in diploid A compared with that in strain B. However, *XYL2* as well as *TKL2* (encoding the minor transketolase) were significantly downregulated in strain A. The sharply decreased NADPH-dependent activity of mutated XR in strain A (Table 1) might be the main reason for the limited xylose flux flow into the non-oxidative PPP. Genes related to gluconeogenesis (*ERR1*, *ERR3*, *PCK1*, and *PYC1*) and acetate formation (*ALD3* and *ALD6*) were downregulated in strain A. The glycerol-producing genes *GPD1* and *GPP2* (encoding glycerol-1-phosphatase) were notably upregulated in response to mutated XR in the diploid strain. The upregulation of these two genes might be due to the higher biomass yield of strain A compared with that of strain B, since more glycerol should be generated to facilitate NADH reoxidation [37].

In summary, there was a significant difference in xylose metabolism between the haploid and diploid strains in response to mutated XR. After expressing NADH-preferring XR in the haploid strain, a lower flux into futile pathways and a higher flux towards ethanol production were indicated, which suggested that the intracellular redox-imbalance was greatly alleviated [8]. By contrast, after expressing NADH-preferring XR in the diploid strain, the flux into ethanol production remained unchanged while the flux into glycerol production increased. This suggested that the redox-imbalance alleviated by expressing mutated XR in diploid was much less than that in haploid. It can be inferred that haploid and diploid might differ greatly in redox homeostasis and regulation. There was no relevant report at present and further study is needed.

### Transcription factors (TFs)

Genes regulation change has long been recognized as an important mechanism for phenotypic evolution. Potential transcription factors (TFs) regulating DEGs were analyzed by YEASTRACT. When comparing HX57D with HX62W, 22 TFs were significantly changed, including one upregulated and 21 downregulated (Fig. 5a, Additional file 1: Table S2). Several enriched TFs (Abf1p, Rme1p, Gat4p, Cup9p, Reb1p, Fkh2p, Hcm1p, and Mot2p) are involved in regulating cell cycle and biosynthesis of protein. Most of them were significantly downregulated, except for Rme1p, the negative regulator of meiosis [39]. It was speculated that altering cofactor preference of XR from NADPH to NADH probably delayed the cell cycle and protein biosynthesis of the haploid, which might lead to the lower biomass yield of HX57D compared with HX62W.

Three TFs (Ixr1p, Cin5p, and Mot3p) are related to response to various stresses, including oxidative stress and osmotic stress [40]. The downregulation of *IXR1*, *CIN5*, and *MOT3* indicated that the oxidative stress was reduced in response to mutated XR in the haploid strain. Moreover, a previous study reported that stress response was associated with xylose utilization [11]. Deletion of *IXR1* was found to increase xylose consumption in aerobic fermentation with glucose and xylose [41].

Thi2p is an activator of thiamine biosynthetic genes. Deletion of *THI2* can promote xylose metabolism when co-fermenting glucose and xylose [27]. Rsf2p is involved in regulating genes required for glycerol-based growth and respiration. Cat8p is necessary for expression of genes involved in gluconeogenesis, respiration, glyoxylic shunt and ethanol utilization. Disrupting *CAT8* improved ethanol production from glucose in *S. cerevisiae* [42] and from xylose in the natural xylose-fermenting yeast *Ogataea polymorpha* [43]. The downregulation of *RSF2* and *CAT8* supported the hypothesis that xylose might be served as fermentative carbon source in response to mutated XR in the haploid strains.

Only eight TFs were significantly changed when comparing diploid A with B (Fig. 5a, Additional file 1: Table S2). Among the upregulated TFs, Gcr1p, a major regulator of glycolytic genes, also activated RNA polymerase II transcription and ribosomal protein biosynthesis [44]; Msa2p involves in regulation of G1-specific transcription and cell cycle initiation. The upregulation of *GCR1* and *MAS1* was associated with an active protein biosynthesis and rapid cell growth of the diploids in response to mutated XR. Among the downregulated TFs, Tog1p and Adr1p are required for non-fermentable carbon metabolism such as ethanol, glycerol, lactate, and fatty acid utilization [45, 46]. Tog1 also involves in oxidative stress tolerance. The downregulation of *TOG1* and *CIN5* suggested a reduced oxidative stress in the diploid strains induced by mutated XR.

In summary, oxidative stress and non-fermentable carbon metabolism related TFs were downregulated in both the haploid and diploid strains in response to changed cofactor preference of XR. Uniquely, TFs associated with cell growth were down-regulated in the haploids and upregulated in the diploids in response to mutated XR. These results were consistent with the fermentation performances and KEGG enrichment results.

### Transcriptional differences between the diploid and haploid strains

To analyze the transcriptional responses of yeast strains to increased ploidy, the transcriptome profile was organized into two comparison groups: A vs. HX57D and B vs. HX62W. A total of 1956 DEGs were found in group A vs. HX57D, including 1042 upregulated and 914 downregulated genes (Additional file 1: Fig. S2c). Meanwhile, 1700 DEGs were found in group B vs. HX62W, including 863 upregulated and 837 downregulated genes (Additional file 1: Fig. S2d). The numbers of the overlapped up- and down-regulated genes in two groups were 497 and 360, respectively (Fig. 2b). These overlapped genes were assumed to be closely related to the different xylose fermentation performances between the diploid and haploid strains, which was further analyzed.

### KEGG pathway analysis

Seven KEGG pathways were enriched for the upregulated genes in the diploids relative to the haploids, including glyoxylate and dicarboxylate metabolism as well as pyruvate metabolism (p < 0.01) (Fig. 3c, Additional file 1: Table S1). Meanwhile, five KEGG pathways were enriched for the downregulated genes, including glycolysis/gluconeogenesis, MAPK signaling pathway-yeast, carbon metabolism as well as fructose and mannose metabolism (p < 0.01) (Fig. 3d, Additional file 1: Table S1). Overall, genes involved in carbohydrate metabolism were significantly affected by increased cell ploidy, which was further discussed later. On the other hand, most DEGs involved in MAPK signaling pathway-yeast were related to mating, and these genes were significantly downregulated in the diploids when fermenting xylose. Similar results were also observed in the diploids during glucose fermentation [18]. Downregulation of mating genes might explain the loss of mating capability in the diploid strains [47].

### Sugar transporters

A large number of transporters were notably changed in response to increased ploidy (Table 2). *HXT4* and *HXT7* are the main transporters during xylose fermentation [29]; *HXT13* is usually induced by non-fermentable carbon source [13]; *HXT17* was identified to transport mannitol, sorbitol and xylitol [48]. The upregulation of *HXT13* and *HXT17* as well as downregulation of *HXT4* and *HXT7* might contribute to the differences in xylose uptake between diploids and haploids.

### Central carbon metabolism

To reveal the effect of increased ploidy on central carbon metabolism, only common DEGs that significantly upregulated or downregulated in two comparison groups (A vs. HX57D and B vs. HX62W) were of interest (Fig. 4). The expression levels of *XYL1*, *XYL2*, and *XKS1* were significantly lower in the diploids, which were consistent with the reduced enzymatic activities of XR and XDH (Table 1). Genes involved in PPP were not responsive to increased ploidy. Moreover, most genes involved in glycolysis (*PGI1*, *FBA1*, *TPI1*, *TDH1*, *TDH2*, *TDH3*, *PGK1*, *GPM1*, *ENO1*, *ENO2*, and *CDC19*) were significantly downregulated in the diploid strains. Additionally, downregulation of *ADH1* and *ADH2* as well as upregulation of *ALD4* (encoding mitochondrial aldehyde dehydrogenase), *ALD6*, and *ACS1* redirected acetaldehyde into the glyoxylate shunt rather than ethanol formation in the diploid strains. Consequently, *ICL1* and *MLS1* (encoding malate synthase) involved in glyoxylate shunt were significantly upregulated in the diploids.

In brief, the diploids had lower carbon fluxes through central carbon metabolism than the haploids when fermenting xylose, and the reduction was more significant in the XR-mutated strains. The fermentation results also confirmed that the xylose consumption rate per gram biomass of strain A was much lower than that of HX57D (data not shown). A previous study reported that the isogenic haploid and diploid strains with native XR were similar in biomass production from xylose [10]. In the present study, the biomass yields of strains B and HX62W were also similar. However, the biomass yield of strain A was much higher than that of HX57D, which might be due to the lower flux through TCA circle in HX57D. This result supported the opinion that biomass yield is inversely correlated with TCA cycle activity [8]. As a result, strain A with a higher biomass yield required more NADH for ATP production instead of ethanol production. Therefore, haploid and diploid strains might have different NADH status. To date, there is no study focusing on the difference in carbon flux between haploid and diploid when using xylose as the sole carbon source. However, the metabolome analysis when glucose was fermented revealed that diploid exhibited higher levels of most glycolytic intermediates than haploid [19]. Therefore, the effects of increased ploidy on carbon metabolism might highly depend on carbon source. The differences in xylose metabolism between diploid and haploid require more systematic and in-depth investigation.

### Transcription factors

A total of 34 TFs were significantly changed when comparing the diploids with the haploids (Fig. 5b, Additional file 1: Table S2). Among them, 9 TFs (Kar4p, Rme1p, Tec1p, Sut1p, Mga1p, Upc2p, Nrg1p, Phd1p, and Nrg2p) are related to sporulation and filamentous growth, which suggested that regulation of cell proliferation and vegetative growth might be two main aspects in response to increased ploidy. In addition, several stress response-related TFs (Cin5p, Cup2p, Nrg1p, and Nrg2p) were significantly upregulated in the diploid strains. A previous study reported that diploid was more tolerant to ethanol, oxidative stress (H_2_O_2_), and metal ions (copper) than haploid [16]. In this study, upregulation of *CIN5*, *CUP2*, *NRG1*, and *NRG2* might be related to the different stress tolerance between the diploids and the haploids.

**Fig. 6:**
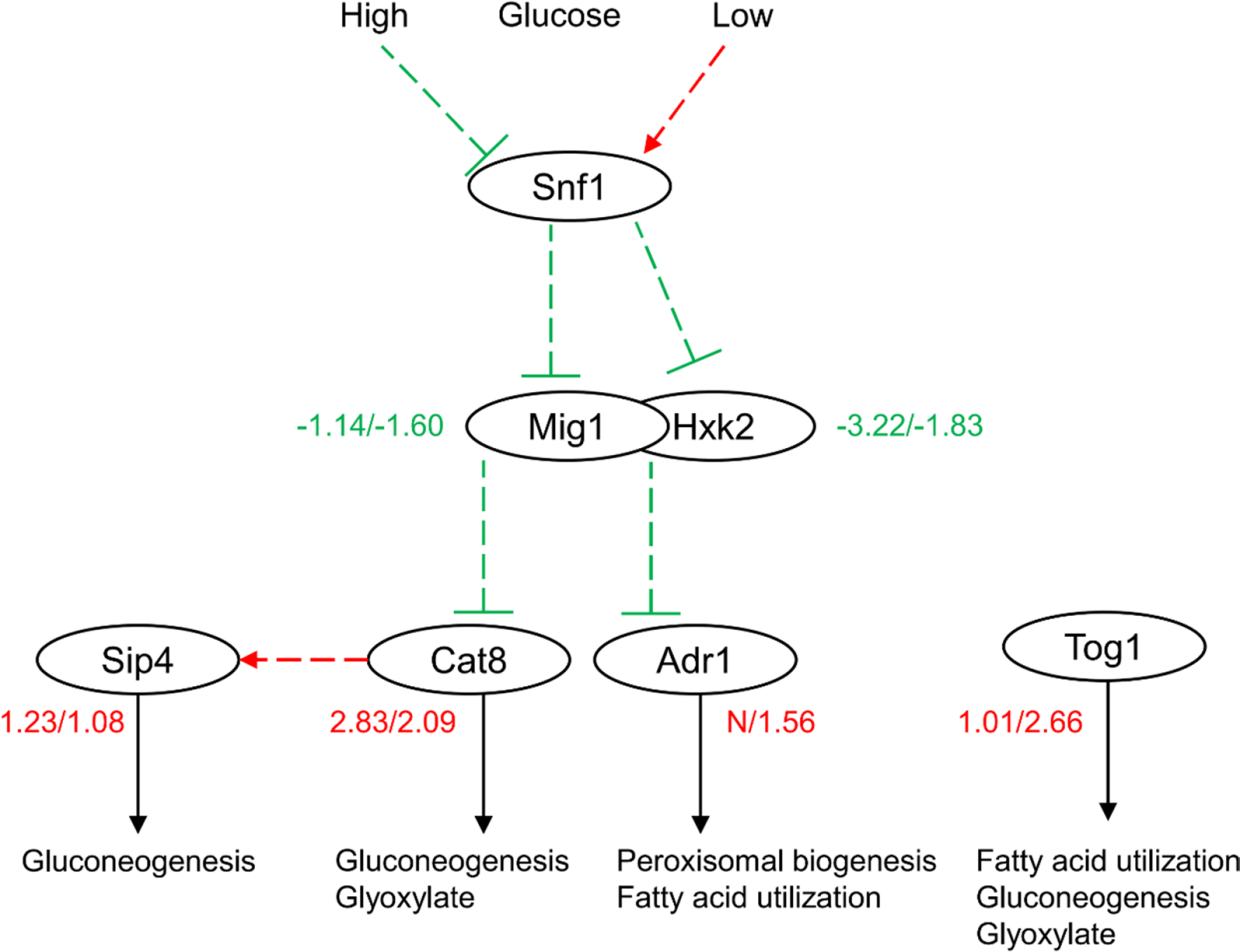
Significantly changed xylose utilizing-related TFs between the diploids and the haploids. Data are presented as log_2_FC for A vs. HX57D (left) and A vs. HX62W (right). Averages of biological triplicates are presented

Moreover, several TFs (Msn4p, Gcr2p, Mig1p, Tog1p, Cat8p, and Sip4p) are related to carbohydrate metabolism. Msn4p and Gcr2p both activate glycolic genes. Downregulation of *MSN4* might have contributed to the reduced carbon flux into glycolysis in the diploids. However, *GCR2* showed increased expression level, and it remained unknown how Gcr2p regulated glycolysis genes during xylose fermentation. The other four TFs (Mig1p, Tog1p, Cat8p, and Sip4p) are related to nonfermentable carbon utilization (Fig. 6). Repressor protein Mig1p is the main TF responsible for glucose repression, while Hxk2p functions as an intracellular glucose sensor as well as an important regulator of glucose repression signal [49]. Respiratory regulator Cat8p, which is repressed by Mig1p, controls the expression of various genes involved in gluconeogenesis and glyoxylate shunt [50]. Increased Cat8p level allows positive regulation of *SIP4*, thereby activating the gluconeogenic genes. Tog1 also functions as an activator of genes involved in fatty acid oxidation, glyoxylate shunt and gluconeogenesis [45]. The noticeable changes in regulation of non-fermentable carbon utilization might contribute to the distinct carbohydrate metabolism in the diploids compared with the haploids (Fig. 6).

In summary, the regulation mechanisms for the xylose metabolism were found to be totally different between diploids and haploids. In a word, xylose was likely to be sensed as a non-fermentative carbon source in the diploids but as a fermentation carbon source in the haploids.

## Conclusions

By changing the cofactor preference of XR from NADPH to NADH, the xylose consumption rate and ethanol yield increased in the haploid strain, while the biomass yield increased in the diploid strain. Comparative transcriptomic analysis revealed that genes involved in heterologous xylose metabolism, PPP, glycolysis, and alcohol fermentation were upregulated in the haploid strain whereas protein biosynthesis related genes were induced in the diploid strain in response to mutated XR, which supported the fermentation results. The effect of mutated XR on xylose metabolism might be largely influenced by the intracellular NADPH homeostasis of the host strains. By comparing the diploids with the haploids, the expression of most genes in central carbon metabolism was repressed and TFs related to non-fermentable carbon utilization and stress resistance were significantly upregulated in the diploids, suggesting xylose was served as a fermentative carbon source in the haploids. This work is the first attempt to characterize the effects of mutated XR and increased ploidy on xylose metabolism at the transcription level in industrial *S. cerevisiae* strain. Our findings provide valuable insights for the construction of lignocellulosic bioethanol producer strain.

## Materials and methods

### Strains and media

All strains used in this study were derivatives of the flocculating industrial *S. cerevisiae* strain KF7 [20] (Table 3). Yeast cells were cultured on YPD20 plates (YP medium (10 g/L yeast extract, 20 g/L peptone) with 20 g/L glucose and 20 g/L agar). Transformants were selected on YPD20 plates supplemented with 100 μg/mL G418 and/or 50 μg/mL nourseothricin. For batch fermentation, yeast cells were pre-cultivated in YPD50 medium (YP medium with 50 g/L glucose), and then fermented in YPX50 medium (YP medium with 50 g/L xylose). *Escherichia coli* DH5α was used for plasmid preparation and cultured in LB medium (10 g/L peptone, 5 g/L yeast extract, 10 g/L NaCl, pH 7.4) supplemented with 100 μg/mL ampicillin, kanamycin or 50 μg/mL X-Gal. Agar (20 g/L) was added in case of plate preparation.

**Table 3:** Strains and plasmids used in this study

Strains and plasmids	Description	Source
*S. cerevisiae* strains		
KF7	*MATa/α*, Flo + , Spo-	[20]
HX62W	KFG4-6B (haploid of KF7), *ura3*::*XYL1*-*XYL2*-*XKS1*-*KanMX*	[21]
HX57D	KFG4-6B, *ura3*::*XYL1*(K270R/N272D)-*XYL2*-*XKS1*-*KanMX*	[21]
KF7Cas9	KF7, Cas9-NAT	This study
A	KF7, *pho13::P*_*TDH3*_*-XYL1*(K270R/N272D)*-P*_*TDH3*_*-XYL2-P*_*TDH3*_*-XKS1*	This study
B	KF7, *pho13*::*P*_*TDH3*_*-XYL1-P*_*TDH3*_*-XYL2-P*_*TDH3*_*-XKS1*	This study
Plasmids		
pXR	*P*_*TDH3*_*-XYL1-T*_*TDH3*_	[52]
pKX1(D)X2XK	*loxP-KanMX-loxP-P*_*TDH3*_*-XYL1*(K270R/N272D)*-T*_*TDH3*_*-P*_*TDH3*_*-XYL2-T*_*TDH3*_*-P*_*TDH3*_*-XKS1-T*_*TDH3*_	[21]
pXDH	*P*_*TDH3*_*-XYL2-T*_*TDH3*_	[52]
pXK	*P*_*TDH3*_*-XKS1-T*_*TDH3*_	[52]
19T-xyl1W	*P*_*TDH3*_*-XYL1-T*_*TDH3*_	This study
1T-xyl1D	*P*_*TDH3*_*-XYL1*(K270R/N272D)*-T*_*TDH3*_	This study
19T-xyl2	*P*_*TDH3*_*-XYL2-T*_*TDH3*_	This study
19T-xks1	*P*_*TDH3*_*-XKS1-T*_*TDH3*_	This study
pKan	*KanMX*	This study
pKan-LacZ	*U*_*PHO13*_*-Aar I-LacZ-Aar I-D*_*PHO13*_	This study
pK-X1(D)-X2-XK	*P*_*TDH3*_*-XYL1*(K270R/N272D)*-T*_*TDH3*_-*P*_*TDH3*_*-XYL2-T*_*TDH3*_-*P*_*TDH3*_*-XKS1-T*_*TDH3*_	This study
pK-X1(W)-X2-XK	*P*_*TDH3*_*-XYL1-T*_*TDH3*_-*P*_*TDH3*_*-XYL2-T*_*TDH3*_-*P*_*TDH3*_*-XKS1-T*_*TDH3*_	This study
pMEL13	2 μm, *ampR*, *KanMX*, *gRNA-CAN1.Y*	[54]
Cas9-NAT	*ampR*, *NAT*, *Cas9*	[55]
pM-gPHO13	pMEL13, *gRNA-PHO13*	This study

### Construction of pK-X1(W)-X2-XK and pK-X1(D)-X2-XK

The plasmids and primers were listed in Table 3 and Additional file 1: Table S3, respectively. Three genes *XYL1*, *XYL2*, and *XKS1* were assembled simultaneously and seamlessly according to the yeast Golden Gate (yGG) method [51] with minor modifications. Native *XYL1* gene and mutated *XYL1* gene were separately amplified using plasmids pXR [52] and pKX1(D)X2XK [21] as the templates with primer set XYL1t-prefix/XYL1t-suffix. *XYL2* gene and *XKS1* gene were amplified using plasmids pXDH and pXK [52] as the templates with primer sets XYL2t-prefix/XYL2t-suffix and XKS1t-prefix/XKS1t-suffix, respectively. These primers included overhangs encoding inwardly facing *Aar*I sites separated by four bases from the appropriate yGG-compatible overhangs. PCR products were cloned into pMD19 to build four donor vectors 19 T-xyl1W, 19 T-xyl1D, 19 T-xyl2, and 19 T-xks1, respectively.

The acceptor vector pKan-LacZ was constructed as follows. Firstly, the backbone of pUC19 and *kanMX* gene were amplified using pUC19 and pET-28a as the templates with primer sets pUC19-F/R and pUC19kan-F/R, respectively. Two PCR products were ligated to generate plasmid pKan through Gibson assembly [53]. Secondly, a *LacZ* expression cassette flanked by outwardly facing *Aar*I sites were amplified from the genome of *E. coli* BL21 using primer set LacZ-F/LacZ-R. The overhangs generated following *Aar*I digestion were compatible with the 5′ overhang of *XYL1* and the 3′ overhang of *XKS1*. Finally, the *LacZ* cassette was inserted into the plasmid pKan to create plasmid pKan-LacZ.

The acceptor vector pKan-LacZ and donor vectors (19 T-xyl1W/D, 19 T-xyl2 and 19 T-xks1) were mixed and assembled using GeneArt Type IIs Assembly Kits (Life technologies, CA, USA) according to the manufacturer’s instructions. Transformants were selected on LB plate containing kanamycin (100 μg/mL) and X-Gal (50 μg/mL). White colonies were selected, and the plasmids were verified by colony PCR. Two plasmids expressing *XYL1*-*XYL2*-*XKS1* and *XYL1*(K270R/N272D)-*XYL2*-*XKS1* cassettes were named as pK-X1(W)-X2-XK and pK-X1(D)-X2-XK, respectively.

### Construction of pM-gPHO13

A specific guide RNA (gRNA) targeting *PHO13* gene was designed by using the Yeastriction tool [54]. The gRNA insert primers were complementary sequences containing 20 bp of target sequences (TTCAACACCGAATTTCATAT) and purchased from Genewiz (Suzhou, China). Two primers PHO13_tgR-F and PHO13_tgR-R were mixed equally and annealed to generate 120 bp of PHO13-gRNA fragment. The backbone of plasmid pMEL13 [54] was amplified with primers 6005 and 6006 and ligated with PHO13-gRNA by Gibson assembly to generate the plasmid pM-gPHO13.

### Strain construction

Gene cassettes *XYL1*(native or mutated)-*XYL2*-*XKS1* were integrated into *PHO13* loci of the diploid KF7 via CRISPR/Cas9 system. KF7 was firstly transformed with plasmid Cas9-NAT [55] to generate strain KF7Cas9. Then, plasmids pK-X1(W)-X2-XK and pK-X1(D)-X2-XK were separately amplified with primers PHO13U and PHO13D, and the PCR products were transformed into KF7Cas9 together with the plasmid pM-gPHO13. Yeast transformation was carried out using a modified lithium acetate method as described previously [54]. Transformants were selected on YPD20 media with G418 and nourseothricin and further checked by colony PCR. Correct transformants were cultured in YPD20 to remove the plasmids Cas9-NAT and pM-gPHO13 using the method described previously [54].

### Xylose fermentation

Yeast cells were aerobically pre-cultivated at 30 °C for 16 h using YPD50 medium. Cells were harvested and inoculated into 100 mL of YPX50 medium (in 300-mL Erlenmeyer flasks with cotton plug) with an initial inoculum size of 4 g/L dry cell weight (DCW). The fermentation was conducted at 35 °C in a thermostatic water bath with an agitation speed of 200 rpm. Samples were periodically taken to analyze the concentrations of cell, sugar, ethanol, and by-products. XR and XDH activities were assessed according to the method described previously [21]. All experiments were performed in triplicate.

Concentrations of xylose and xylitol were determined by HPLC equipped with a RID-10A refractive index detector (Shimadzu, Tokyo, Japan) [56]. Concentration of ethanol was measured by GC353B with a FID detector, and isopropanol was used as the internal standard [56]. DCW was determined according to the method described previously [57].

### Transcriptome analysis

Total RNA was extracted from cells collected at 8 h of fermentation using the Yeast RNA Kit (Omega Bio-Tek, GA, USA) according to the manufacturer’s instructions. Three biological replicated fermentation and RNA-seq analysis were performed independently. RNA degradation and contamination were monitored by agarose gel electrophoresis. RNA purity was tested using the NanoPhotometer® spectrophotometer (Implen, CA, USA). RNA concentration was measured using the Qubit® 2.0 Fluorimeter (Life Technologies, CA, USA). RNA integrity was evaluated using the Bioanalyzer 2100 system (Agilent Technologies, CA, USA).

The RNA-Seq library was prepared and sequenced on an Illumina HiSeq X Ten platform at Novogene Technology Co. Ltd. (Beijing, China) according to the method described previously [58]. 4 G clean data were obtained for each sample. The raw sequence data can be accessed through the SRA accession PRJNA556802. The comparative transcriptome was analyzed according to the procedures described previously [59]. Fragments per kilobase of exon per million reads mapped (FPKM) was used for estimating gene expression levels. Differentially expressed genes (DEGs) were screened out with a threshold of false discovery rate (FDR) < 0.05 and an absolute log_2_fold change (FC) ≥ 1. Gene functions were annotated based on the *Saccharomyces* genome database (SGD) (https://www.yeastgenome.org/). KEGG pathways were retrieved from KEGG database and enriched using KOBAS. KEGG pathways with a *p* < 0.05 were considered significantly enriched. The enrichment ratio of each KEGG pathway was the number of DEGs involved in each KEGG pathway to the number of total genes in each pathway. Transcription factors (TFs) were identified by using YEASTRACT database. The enrichment ratio of each TF was the number of DEGs regulated by the TF to the number of total DEGs.

### Quantitative reverse-transcription PCR (qRT-PCR)

The cDNA was reverse-transcribed from total RNA using the PrimeScript™ RT reagent Kit with gDNA Eraser (Takara, Dalian, China). qRT-PCR analysis was performed using TB Green™ Premix Ex Taq™ II (Tli RNaseH Plus) (Takara, Dalian, China). *ACT1* was served as the normalization standard. The primers used were listed in Additional file 1: Table S3. Triplicate assays were carried out for each sample.

## Supplementary information

**Additional file 1: Table S1.** Enriched KEGG pathways. **Table S2.** Description of transcription factors. **Table S3.** Primers used in this study. **Fig. S1.** Validation of transcriptome data by real-time qRT-PCR. Fold change (FC) is the ratio of transcription level of specific gene in experimental group to that in control. *ACT1* was used as a reference gene. **Fig. S2.** Cluster analysis of DEGs involved in comparison groups (a) HX57D vs. HX62W; (b) A vs. B; (c) A vs. HX57D; and (d) B vs. HX62W. Three biological replicates were carried out for each sample.

## Data Availability

The transcriptome datasets analyzed in this study can be accessed through the SRA accession PRJNA556802.

## Abbreviations

XR: Xylose reductase
XDH: Xylitol dehydrogenase
XK: Xylulokinase
XI: Xylose isomerase
DCW: Dry cell weight
DEGs: Differentially expressed genes
FPKM: Fragments per kilobase of exon per million reads mapped
FC: Fold change
TFs: Transcription factors
TCA: Tricarboxylic acid
PPP: Pentose phosphate pathway
qRT-PCR: Quantitative reverse-transcription polymerase chain reaction
gRNA: Guide RNA
SGD: *Saccharomyces* Genome database
yGG: Yeast Golden Gate
FDR: False discovery rate

## References

[1] Kwak S, Jin YS (2017). Production of fuels and chemicals from xylose by engineered *Saccharomyces cerevisiae*: a review and perspective. Microb Cell Fact.

[2] Karhumaa K, Garcia Sanchez R, Hahn-Hagerdal B, Gorwa-Grauslund MF (2007). Comparison of the xylose reductase-xylitol dehydrogenase and the xylose isomerase pathways for xylose fermentation by recombinant *Saccharomyces cerevisiae*. Microb Cell Fact.

[3] Li YC, Xie CY, Yang BX, Tang YQ, Wu B, Sun ZY (2019). Comparative transcriptome analysis of recombinant industrial *Saccharomyces cerevisiae* strains with different xylose utilization pathways. Appl Biochem Biotechnol.

[4] Watanabe S, Pack SP, Saleh AA, Annaluru N, Kodaki T, Makino K (2007). The positive effect of the decreased NADPH-preferring activity of xylose reductase from *Pichia stipitis* on ethanol production using xylose-fermenting recombinant *Saccharomyces cerevisiae*. Biosci Biotech Bioch.

[5] Watanabe S, Abu Saleh A, Pack SP, Annaluru N, Kodaki T, Makino K (2007). Ethanol production from xylose by recombinant *Saccharomyces cerevisiae* expressing protein-engineered NADH-preferring xylose reductase from *Pichia stipitis*. Microbiology.

[6] Bengtsson O, Hahn-Hägerdal B, Gorwa-Grauslund MF (2009). Xylose reductase from *Pichia stipitis* with altered coenzyme preference improves ethanolic xylose fermentation by recombinant *Saccharomyces cerevisiae*. Biotechnol Biofuels.

[7] Runquist D, Hahn-Hägerdal B, Bettiga M (2010). Increased ethanol productivity in xylose-utilizing *Saccharomyces cerevisiae* via a randomly mutagenized xylose reductase. Appl Environ Microbiol.

[8] Feng X, Zhao H (2013). Investigating xylose metabolism in recombinant *Saccharomyces cerevisiae* via ^13^C metabolic flux analysis. Microb Cell Fact.

[9] Feng X, Zhao H (2013). Investigating host dependence of xylose utilization in recombinant *Saccharomyces cerevisiae* strains using RNA-seq analysis. Biotechnol Biofuels.

[10] Lopes DD, Rosa CA, Hector RE, Dien BS, Mertens JA, Ayub MAZ (2017). Influence of genetic background of engineered xylose-fermenting industrial *Saccharomyces cerevisiae* strains for ethanol production from lignocellulosic hydrolysates. J Ind Microbiol Biotechnol.

[11] Cheng C, Tang RQ, Xiong L, Hector RE, Bai FW, Zhao XQ (2018). Association of improved oxidative stress tolerance and alleviation of glucose repression with superior xylose-utilization capability by a natural isolate of *Saccharomyces cerevisiae*. Biotechnol Biofuels.

[12] Garay-Arroyo A, Covarrubias AA, Clark I, Niño I, Gosset G, Martinez A (2004). Response to different environmental stress conditions of industrial and laboratory *Saccharomyces cerevisiae* strains. Appl Microbiol Biotechnol.

[13] Matsushika A, Goshima T, Hoshino T (2014). Transcription analysis of recombinant industrial and laboratory *Saccharomyces cerevisiae* strains reveals the molecular basis for fermentation of glucose and xylose. Microb Cell Fact.

[14] Matsushika A, Inoue H, Murakami K, Takimura O, Sawayama S (2009). Bioethanol production performance of five recombinant strains of laboratory and industrial xylose-fermenting *Saccharomyces cerevisiae*. Bioresour Technol.

[15] Yamada R, Tanaka T, Ogino C, Fukuda H, Kondo A (2010). Novel strategy for yeast construction using *delta*-integration and cell fusion to efficiently produce ethanol from raw starch. Appl Microbiol Biotechnol.

[16] Zhang K, Fang YH, Gao KH, Sui Y, Zheng DQ, Wu XC (2017). Effects of genome duplication on phenotypes and industrial applications of *Saccharomyces cerevisiae* strains. Appl Microbiol Biotechnol.

[17] Katou T, Kitagaki H, Akao T, Shimoi H (2008). Brewing characteristics of haploid strains isolated from sake yeast Kyokai No. 7. Yeast..

[18] Li BZ, Cheng JS, Ding MZ, Yuan YJ (2010). Transcriptome analysis of differential responses of diploid and haploid yeast to ethanol stress. J Biotechnol.

[19] Ding MZ, Li BZ, Cheng JS, Yuan YJ (2010). Metabolome analysis of differential responses of diploid and haploid yeast to ethanol stress. OMICS.

[20] Kida K, Kume K, Morimura S, Sonoda Y (1992). Repeated-batch fermentation process using a thermotolerant flocculating yeast constructed by protoplast fusion. J Ferment Bioeng.

[21] Xie CY, Yang BX, Wu YJ, Xia ZY, Gou M, Sun ZY (2020). Construction of industrial xylose-fermenting *Saccharomyces cerevisiae* strains through combined approaches. Process Biochem.

[22] Karhumaa K, Fromanger R, Hahn-Hagerdal B, Gorwa-Grauslund MF (2007). High activity of xylose reductase and xylitol dehydrogenase improves xylose fermentation by recombinant *Saccharomyces cerevisiae*. Appl Microbiol Biotechnol.

[23] Zha J, Shen M, Hu M, Song H, Yuan Y (2014). Enhanced expression of genes involved in initial xylose metabolism and the oxidative pentose phosphate pathway in the improved xylose-utilizing *Saccharomyces cerevisiae* through evolutionary engineering. J Ind Microbiol Biotechnol.

[24] Mohammad K, Dakik P, Medkour Y, McAuley M, Mitrofanova D, Titorenko VI (2018). Some metabolites act as second messengers in yeast chronological aging. Int J Mol Sci..

[25] Duenas-Sanchez R, Gutierrez G, Rincon AM, Codon AC, Benitez T (2012). Transcriptional regulation of fermentative and respiratory metabolism in *Saccharomyces cerevisiae* industrial bakers' strains. FEMS Yeast Res.

[26] Regenberg B, Grotkjaer T, Winther O, Fausbøll A, Akesson M, Bro C (2006). Growth-rate regulated genes have profound impact on interpretation of transcriptome profiling in *Saccharomyces cerevisiae*. Genome Biol.

[27] Wei S, Liu Y, Wu M, Ma T, Bai X, Hou J (2018). Disruption of the transcription factors Thi2p and Nrm1p alleviates the post-glucose effect on xylose utilization in *Saccharomyces cerevisiae*. Biotechnol Biofuels.

[28] Hamacher T, Becker J, Gardonyi M, Hahn-Hagerdal B, Boles E (2002). Characterization of the xylose-transporting properties of yeast hexose transporters and their influence on xylose utilization. Microbiology.

[29] Han JH, Park JY, Yoo KS, Kang HW, Choi GW, Chung BW (2011). Effect of glucose on xylose utilization in *Saccharomyces cerevisiae* harboring the xylose reductase gene. Arch Microbiol.

[30] Diderich JA, Schuurmans JM, Van Gaalen MC, Kruckeberg AL, Van Dam K (2001). Functional analysis of the hexose transporter homologue *HXT5* in *Saccharomyces cerevisiae*. Yeast.

[31] Goncalves DL, Matsushika A, de Sales BB, Goshima T, Bon EP, Stambuk BU (2014). Xylose and xylose/glucose co-fermentation by recombinant *Saccharomyces cerevisiae* strains expressing individual hexose transporters. Enzyme Microb Technol.

[32] Li YC, Gou ZX, Liu ZS, Tang YQ, Akamatsu T, Kida K (2014). Synergistic effects of *TAL1* over-expression and *PHO13* deletion on the weak acid inhibition of xylose fermentation by industrial *Saccharomyces cerevisiae* strain. Biotechnol Lett.

[33] Matsushika A, Nagashima A, Goshima T, Hoshino T (2013). Fermentation of xylose causes inefficient metabolic state due to carbon/energy starvation and reduced glycolytic flux in recombinant industrial *Saccharomyces cerevisiae*. PLoS ONE.

[34] Shen Y, Chen X, Peng B, Chen L, Hou J, Bao X (2012). An efficient xylose-fermenting recombinant *Saccharomyces cerevisiae* strain obtained through adaptive evolution and its global transcription profile. Appl Microbiol Biotechnol.

[35] Bakker BM, Bro C, Kotter P, Luttik MA, van Dijken JP, Pronk JT (2000). The mitochondrial alcohol dehydrogenase Adh3p is involved in a redox shuttle in *Saccharomyces cerevisiae*. J Bacteriol.

[36] Zeng WY, Tang YQ, Gou M, Sun ZY, Xia ZY, Kida K (2017). Comparative transcriptomes reveal novel evolutionary strategies adopted by *Saccharomyces cerevisiae* with improved xylose utilization capability. Appl Microbiol Biotechnol.

[37] Vandijken J, Scheffers W (1986). Redox balances in the metabolism of sugars by yeasts. FEMS Microbiol Lett.

[38] Zhao Y, Liu M, He L, Li X, Wang F, Yan B (2019). A cytosolic NAD(+)-dependent GPDH from maize (ZmGPDH1) is involved in conferring salt and osmotic stress tolerance. BMC Plant Biol.

[39] van Dyk D, Hansson G, Pretorius IS, Bauer FF (2003). Cellular differentiation in response to nutrient availability: The repressor of meiosis, Rme1p, positively regulates invasive growth in *Saccharomyces cerevisiae*. Genetics.

[40] Castro-Prego R, Lamas-Maceiras M, Soengas P, Carneiro I, Gonzalez-Siso I, Cerdan ME (2009). Regulatory factors controlling transcription of *Saccharomyces cerevisiae IXR1* by oxygen levels: a model of transcriptional adaptation from aerobiosis to hypoxia implicating *ROX1* and *IXR1* cross-regulation. Biochem J.

[41] Wei S, Bai P, Liu Y, Yang M, Ma J, Hou J (2019). A Thi2p regulatory network controls the post-glucose effect of xylose utilization in *Saccharomyces cerevisiae*. Front Microbiol.

[42] Michael DG, Maier EJ, Brown H, Gish SR, Fiore C, Brown RH (2016). Model-based transcriptome engineering promotes a fermentative transcriptional state in yeast. P Natl Acad Sci USA.

[43] Ruchala J, Kurylenko OO, Soontorngun N, Dmytruk KV, Sibirny AA (2017). Transcriptional activator Cat8 is involved in regulation of xylose alcoholic fermentation in the thermotolerant yeast *Ogataea* (*Hansenula*) *polymorpha*. Microb Cell Fact.

[44] Barbara KE, Haley TM, Willis KA, Santangelo GM (2007). The transcription factor Gcr1 stimulates cell growth by participating in nutrient-responsive gene expression on a global level. Mol Genet Genomics.

[45] Thepnok P, Ratanakhanokchai K, Soontorngun N (2014). The novel zinc cluster regulator Tog1 plays important roles in oleate utilization and oxidative stress response in *Saccharomyces cerevisiae*. Biochem Biophys Res Commun.

[46] Manzanares-Estreder S, Espi-Bardisa J, Alarcon B, Pascual-Ahuir A, Proft M (2017). Multilayered control of peroxisomal activity upon salt stress in *Saccharomyces cerevisiae*. Mol Microbiol.

[47] Xie ZX, Mitchell LA, Liu HM, Li BZ, Liu D, Agmon N (2018). Rapid and efficient CRISPR/Cas9-based mating-type switching of *Saccharomyces cerevisiae*. G3 (Bethesda).

[48] Jordan P, Choe JY, Boles E, Oreb M (2016). Hxt13, Hxt15, Hxt16 and Hxt17 from *Saccharomyces cerevisiae* represent a novel type of polyol transporters. Sci Rep.

[49] Lin Y, Chomvong K, Acosta-Sampson L, Estrela R, Galazka JM, Kim SR (2014). Leveraging transcription factors to speed cellobiose fermentation by *Saccharomyces cerevisiae*. Biotechnol Biofuels.

[50] Turcotte B, Liang XB, Robert F, Soontorngun N (2010). Transcriptional regulation of nonfermentable carbon utilization in budding yeast. FEMS Yeast Res.

[51] Agmon N, Mitchell LA, Cai Y, Ikushima S, Chuang J, Zheng A (2015). Yeast golden gate (yGG) for the efficient assembly of *S. cerevisiae* transcription units. ACS Synth Biol..

[52] Tomitaka M, Taguchi H, Fukuda K, Akamatsu T, Kida K (2013). Isolation and characterization of a mutant recombinant *Saccharomyces cerevisiae* strain with high efficiency xylose utilization. J Biosci Bioeng.

[53] Gibson DG (2012). Oligonucleotide assembly in yeast to produce synthetic DNA fragments. Methods Mol Biol.

[54] Mans R, van Rossum HM, Wijsman M, Backx A, Kuijpers NG, van den Broek M (2015). CRISPR/Cas9: a molecular Swiss army knife for simultaneous introduction of multiple genetic modifications in *Saccharomyces cerevisiae*. FEMS Yeast Res..

[55] Zhang GC, Kong II, Kim H, Liu JJ, Cate JH, Jin YS (2014). Construction of a quadruple auxotrophic mutant of an industrial polyploid *Saccharomyces cerevisiae* strain by using RNA-guided Cas9 nuclease. Appl Environ Microbiol.

[56] Tang Y, An M, Liu K, Nagai S, Shigematsu T, Morimura S (2006). Ethanol production from acid hydrolysate of wood biomass using the flocculating yeast *Saccharomyces cerevisiae* strain KF-7. Process Biochem.

[57] Sonderegger M, Jeppsson M, Larsson C, Gorwa-Grauslund MF, Boles E, Olsson L (2004). Fermentation performance of engineered and evolved xylose-fermenting *Saccharomyces cerevisiae* strains. Biotechnol Bioeng.

[58] Zhang C, Li Z, Zhang X, Yuan L, Dai H, Xiao W (2016). Transcriptomic profiling of chemical exposure reveals roles of Yap1 in protecting yeast cells from oxidative and other types of stresses. Yeast.

[59] Li YC, Zeng WY, Gou M, Sun ZY, Xia ZY, Tang YQ (2017). Transcriptome changes in adaptive evolution of xylose-fermenting industrial *Saccharomyces cerevisiae* strains with δ-integration of different xylA genes. Appl Microbiol Biotechnol.

