# Supplementary material for: Different transcriptional responses of haploid and diploid S. cerevisiae strains to changes in cofactor preference of XR
Source: Microb Cell Fact. 2020 Nov 13;19:211. doi: 10.1186/s12934-020-01474-2 (PMC7666519; doi:10.1186/s12934-020-01474-2)
Supplement: Supplementary file 1 — Additional file 1: Table S1. Enriched KEGG pathways. Table S2. Description of transcription factors. Table S3. Primers used in this study. Fig. S1. Validation of transcriptome data by real-time qRT-PCR. Fold change (FC) is the ratio of transcription level of specific gene in experimental group to that in control. ACT1 was used as a reference gene. Fig. S2. Cluster analysis of DEGs involved in comparison groups (a) HX57D vs. HX62W; (b) A vs. B; (c) A vs. HX57D; and (d) B vs. HX62W. Three biological replicates were carried out for each sample. [file 12934_2020_1474_MOESM1_ESM.docx]

**Different transcriptional responses of haploid and diploid *S. cerevisiae* strains to changes in cofactor preference of XR**

Cai-Yun Xie, Bai-Xue Yang, Qing-Ran Song, Zi-Yuan Xia, Min Gou*, Yue-Qin Tang*

**Additional file 1 - content**

**Table S1** Enriched KEGG pathways

**Table S2** Description of transcription factors

**Table S3** Primers used in this study

**Fig. S1** Validation of transcriptome data by real-time qRT-PCR. Fold change (FC) is the ratio of transcription level of specific gene in experimental group to that in control. *ACT1* was used as a reference gene.

**Fig. S2** Cluster analysis of DEGs involved in comparison groups (**a**) HX57D vs. HX62W; (**b**) A vs. B; (**c**) A vs. HX57D; and (**d**) B vs. HX62W. Three biological replicates were carried out for each sample.

**Table S1** Enriched KEGG pathways

| **ID** | **Term** | ***P* value** | **Genes** |
| --- | --- | --- | --- |
| **HX57D vs. HX62W (p <0.02)** | |  |  |
| sce01200 | Carbon metabolism | 6.92E-07 | *PYC2*, *MET13*, *SHH4*, *FBP1*, *YJL068C*, *GPM2*, *HXK2*, *PGK1*, *TDH1*, *ENO2*, *CDC19*, *IRC15*, *DAL7*, *PCK1*, *TDH2*, *TDH3*, *IDP2*, *TPI1*, *IDH2*, *GPM1*, *GND1*, *FBA1*, *DAK2*, *GCV2*, *ENO1*, *FDH1*, *SOL3*, *TAL1*, *ACS1*, *IDP3*, *FDH2*, *GND2*, *ICL1* |
| sce00010 | Glycolysis / Gluconeogenesis | 1.00E-06 | *TDH2*, *GPM2*, *ALD6*, *YMR099C*, *HXK2*, *PGK1*, *TDH1*, *ENO2*, *CDC19*, *PCK1*, *FBP1*, *TDH3*, *ADH1*, *TPI1*, *GPM1*, *FBA1*, *ENO1*, *ADH2*, *PRM15*, *ADH6*, *PDC1*, *ACS1*, *IRC15* |
| sce00040 | Pentose and glucuronate interconversions | 1.83E-04 | *XYL2*, *GRE3*, *ADH6*, *SOR1*, *XYL1*, *XKS1*, *ALD6*, *UGP1*, *SOR2* |
| sce00680 | Methane metabolism | 3.00E-04 | *ENO2*, *FBP1*, *ACS1*, *GPM1*, *GPM2*, *FBA1*, *FDH2*, *DAK2*, *YJL068C*, *ENO1*, *FDH1* |
| sce01110 | Biosynthesis of secondary metabolites | 1.15E-03 | *SHH4*, *INM2*, *TDH2*, *POT1*, *GPM2*, *ALD6*, *YMR099C*, *HXK2*, *PGK1*, *UGP1*, *GPH1*, *TDH1*, *ENO2*, *CDC19*, *IRC15*, *IDP2*, *PCK1*, *ASP3-3*, *FBP1*, *GAD1*, *CTA1*, *TDH3*, *LEU2*, *ASP3-4*, *ADH1*, *TPI1*, *LYS9*, *IDH2*, *PRM15*, *GND1*, *FBA1*, *INO1*, *YDL086W*, *ENO1*, *ADH2*, *ARO9*, *GPM1*, *SOL3*, *ADH6*, *ERG12*, *INM1*, *PDC1*, *BTS1*, *TAL1*, *ACS1*, *IDP3*, *GND2*, *ARG5,6* |
| sce00051 | Fructose and mannose metabolism | 4.40E-03 | *XYL2*, *SOR1*, *FBP1*, *MAN2*, *TPI1*, *SOR2*, *HXK2*, *FBA1*, *DSF1* |
| sce01230 | Biosynthesis of amino acids | 1.45E-02 | *PYC2*, *TDH2*, *GPM2*, *ARG5,6*, *PGK1*, *PRO2*, *TDH1*, *ENO2*, *CDC19*, *GLN1*, *TDH3*, *LEU2*, *IDP2*, *TPI1*, *IDH2*, *GPM1*, *LYS9*, *FBA1*, *YHR033W*, *ENO1*, *TAL1*, *LYS4*, *IDP3* |
| sce00630 | Glyoxylate and dicarboxylate metabolism | 1.94E-02 | *DAL7*, *CTA1*, *GLN1*, *FDH2*, *ICL1*, *FDH1* |
| **A vs. B (p <0.04)** | |  |  |
| sce00040 | Pentose and glucuronate interconversions | 3.97E-03 | *XYL1*, *XYL2*, *SOR2*, *SOR1*, *ALD6* |
| sce03020 | RNA polymerase | 5.02E-03 | *RPA49*, *RPA43*, *RPC40*, *RPC25*, *RET1*, *RPA12*, *RPC19* |
| sce00052 | Galactose metabolism | 5.87E-03 | *MAL12*, *MAL32*, *IMA4*, *SUC2*, *IMA2*, *IMA3* |
| sce03010 | Ribosome | 7.86E-03 | *RPL22A*, *RPL2B*, *RPL21A*, *RPL25*, *RPS6B*, *RPL9A*, *RPS9B*, *RPS4A*, *RPL14A*, *RPL7A*, *RPL28*, *RPL22B*, *RPS12*, *RPS22A*, *RPS1B*, *RPL8A*, *RPS8B*, *RPL11B*, *RPL12B*, *RPS6A*, *RPL15A* |
| sce00500 | Starch and sucrose metabolism | 1.86E-02 | *MAL12*, *MAL32*, *GSC2*, *IMA4*, *SUC2*, *IMA2*, *IMA3* |
| sce00630 | Glyoxylate and dicarboxylate metabolism | 1.88E-02 | *FDH2*, *CTT1*, *CTA1*, *FDH1* |
| sce03040 | Spliceosome | 3.46E-02 | *SSA1*, *SNU13*, *PRP43*, *SAD1*, *CEF1*, *DBP2*, *FAL1*, *THO2*, *SSA4*, *SSA2* |
| sce03008 | Ribosome biogenesis in eukaryotes | 3.57E-02 | *UTP15*, *SNU13*, *NOP56*, *NHP2*, *NOG1*, *UTP8*, *UTP18*, *IMP4*, *UTP6*, *UTP9*, *NOP1* |
| **Up-regulated genes between diploid strains and haploid strains (p <0.01)** | | | |
| sce00640 | Propanoate metabolism | 1.33E-03 | *ACC1*, *EHD3*, *PDH1*, *ACS1*, *LSC2*, *GRE2* |
| sce01212 | Fatty acid metabolism | 1.33E-03 | *ACC1*, *POX1*, *POT1*, *OLE1*, *FAS2*, *FAA1*, *FAA2*, *ELO1* |
| sce00380 | Tryptophan metabolism | 5.72E-03 | *CTT1*, *CTA1*, *ALD4*, *ALD6*, *BNA4*, *BNA3* |
| sce00630 | Glyoxylate and dicarboxylate metabolism | 6.00E-03 | *CTT1*, *CTA1*, *TDA10*, *MLS1*, *FDH2*, *ICL1*, *YPL276W* |
| sce00270 | Cysteine and methionine metabolism | 6.22E-03 | *STR3*, *YLL058W*, *CYS3*, *ADI1*, *MET17*, *SAM2*, *MET2*, *MET6*, *SAM4* |
| sce00071 | Fatty acid degradation | 7.24E-03 | *POX1*, *POT1*, *ALD4*, *ALD6*, *FAA1*, *FAA2* |
| sce00620 | Pyruvate metabolism | 7.26E-03 | *PYC1*, *ACC1*, *DLD1*, *ACS1*, *MLS1*, *ALD4*, *ALD6*, *CYB2*, *GRE2* |
| **Down-regulated genes between diploid strains and haploid strains (p <0.01)** | | | |
| sce00010 | Glycolysis / Gluconeogenesis | 5.99E-05 | *ENO2*, *ADH1*, *TDH3*, *CDC19*, *TDH2*, *PGI1*, *TPI1*, *GPM1*, *FBA1*, *PGK1*, *ENO1*, *HXK2*, *ADH2*, *TDH1* |
| sce04011 | MAPK signaling pathway - yeast | 2.09E-03 | *STE18*, *GPA1*, *STE5*, *MFA1*, *STE2*, *FUS3*, *MSN4*, *FAR1*, *TEC1*, *MFA2*, *FKS1* |
| sce01230 | Biosynthesis of amino acids | 4.47E-03 | *ENO2*, *TDH3*, *SRG1*, *GLY1*, *TDH2*, *GLN1*, *TPI1*, *ARO1*, *GPM1*, *SER3*, *LYS20*, *FBA1*, *IRC7*, *PGK1*, *ENO1*, *CDC19*, *TDH1* |
| sce01200 | Carbon metabolism | 8.23E-03 | *ENO2*, *TDH3*, *SRG1*, *CDC19*, *TDH2*, *PGI1*, *TPI1*, *GPM1*, *SER3*, *FBA1*, *PGK1*, *YJL045W*, *ENO1*, *HXK2*, *TDH1* |
| sce00051 | Fructose and mannose metabolism | 9.70E-03 | *XYL2*, *TPI1*, *SEC53*, *HXK2*, *FBA1*, *PSA1* |

**Table S2** Description of transcription factors

| TFs | Description |
| --- | --- |
| Cbf1p | Dual function helix-loop-helix protein; binds the motif CACRTG present at several sites including MET gene promoters and centromere DNA element I (CDEI); affects nucleosome positioning at this motif; associates with other transcription factors such as Met4p and Isw1p to mediate transcriptional activation or repression; associates with kinetochore proteins and required for efficient chromosome segregation; protein abundance increases in response to DNA replication stress |
| Met32p | Zinc-finger DNA-binding protein, involved in transcriptional regulation of the methionine biosynthetic genes, similar to Met31p |
| Tec1p | Transcription factor targeting filamentation genes and Ty1 expression; positive regulator of chronological life span; TEA/ATTS DNA-binding domain family member |
| Msn4p | Stress-responsive transcriptional activator; activated in stochastic pulses of nuclear localization in response to various stress conditions; binds DNA at stress response elements of responsive genes, inducing gene expression |
| Gcr2p | Transcriptional activator of genes involved in glycolysis; interacts and functions with the DNA-binding protein Gcr1p |
| Cin5p | Basic leucine zipper (bZIP) transcription factor of the yAP-1 family; physically interacts with the Tup1-Cyc8 complex and recruits Tup1p to its targets; mediates pleiotropic drug resistance and salt tolerance; nuclearly localized under oxidative stress and sequestered in the cytoplasm by Lot6p under reducing conditions |
| Kar4p | Transcription factor required for gene regulation in response to pheromones; also required during meiosis; exists in two forms, a slower-migrating form more abundant during vegetative growth and a faster-migrating form induced by pheromone |
| Sut1p | Transcription factor of the Zn[II]2Cys6 family involved in sterol uptake; involved in induction of hypoxic gene expression |
| Rme1p | Zinc finger protein involved in control of meiosis; prevents meiosis by repressing IME1 expression and promotes mitosis by activating CLN2 expression; directly repressed by a1-alpha2 regulator; mediates cell type control of sporulation |
| Cup2p | Copper-binding transcription factor; activates transcription of the metallothionein genes CUP1-1 and CUP1-2 in response to elevated copper concentrations |
| Mig1p | Transcription factor involved in glucose repression; sequence specific DNA binding protein containing two Cys2His2 zinc finger motifs; regulated by the SNF1 kinase and the GLC7 phosphatase |
| Mga1p | Protein similar to heat shock transcription factor; multicopy suppressor of pseudohyphal growth defects of ammonium permease mutants |
| Upc2p | Sterol regulatory element binding protein, induces transcription of sterol biosynthetic genes and of DAN/TIR gene products; Ecm22p homolog; relocates from intracellular membranes to perinuclear foci on sterol depletion |
| Opi1p | Transcriptional regulator of a variety of genes; phosphorylation by protein kinase A stimulates Opi1p function in negative regulation of phospholipid biosynthetic genes; involved in telomere maintenance |
| Mal33p | MAL-activator protein, part of complex locus MAL3; nonfunctional in genomic reference strain S288C |
| Nrg1p | Transcriptional repressor that recruits the Cyc8p-Tup1p complex to promoters; mediates glucose repression and negatively regulates a variety of processes including filamentous growth and alkaline pH response |
| Tog1p | Putative zinc cluster protein; deletion confers sensitivity to Calcufluor white, and prevents growth on glycerol or lactate as sole carbon source |
| Hap5p | Subunit of the heme-activated, glucose-repressed Hap2/3/4/5 CCAAT-binding complex, a transcriptional activator and global regulator of respiratory gene expression; required for assembly and DNA binding activity of the complex |
| Phd1p | Transcriptional activator that enhances pseudohyphal growth; physically interacts with the Tup1-Cyc8 complex and recruits Tup1p to its targets; regulates expression of FLO11, an adhesin required for pseudohyphal filament formation; similar to StuA, an A. nidulans developmental regulator; potential Cdc28p substrate |
| Cat8p | Zinc cluster transcriptional activator necessary for derepression of a variety of genes under non-fermentative growth conditions, active after diauxic shift, binds carbon source responsive elements |
| YGR067C | Putative protein of unknown function; contains a zinc finger motif similar to that of Adr1p |
| Rpi1p | Putative transcriptional regulator; overexpression suppresses the heat shock sensitivity of wild-type RAS2 overexpression and also suppresses the cell lysis defect of an mpk1 mutation |
| Rtg1p | Transcription factor (bHLH) involved in interorganelle communication between mitochondria, peroxisomes, and nucleus |
| Gal80p | Transcriptional regulator involved in the repression of GAL genes in the absence of galactose; inhibits transcriptional activation by Gal4p; inhibition relieved by Gal3p or Gal1p binding |
| Stp4p | Protein containing a Kruppel-type zinc-finger domain; has similarity to Stp1p, Stp2p, and Stp3p |
| Nrg2p | Transcriptional repressor that mediates glucose repression and negatively regulates filamentous growth; has similarity to Nrg1p |
| Sip4p | C6 zinc cluster transcriptional activator that binds to the carbon source-responsive element (CSRE) of gluconeogenic genes; involved in the positive regulation of gluconeogenesis; regulated by Snf1p protein kinase; localized to the nucleus |
| Dal80p | Negative regulator of genes in multiple nitrogen degradation pathways; expression is regulated by nitrogen levels and by Gln3p; member of the GATA-binding family, forms homodimers and heterodimers with Deh1p |
| Cha4p | DNA binding transcriptional activator, mediates serine/threonine activation of the catabolic L-serine (L-threonine) deaminase (CHA1); Zinc-finger protein with Zn[2]-Cys[6] fungal-type binuclear cluster domain |
| Tbs1p | Putative protein of unknown function; the authentic, non-tagged protein is detected in highly purified mitochondria in high-throughput studies |
| Tod6p | PAC motif binding protein involved in rRNA and ribosome biogenesis; subunit of the RPD3L histone deacetylase complex; Myb-like HTH transcription factor, similar to Dot6p; hypophosphorylated by rapamycin treatment in a Sch9p-dependent manne |
| Gal3p | Transcriptional regulator involved in activation of the GAL genes in response to galactose; forms a complex with Gal80p to relieve Gal80p inhibition of Gal4p; binds galactose and ATP but does not have galactokinase activity |
| Gsm1p | Putative zinc cluster protein of unknown function; proposed to be involved in the regulation of energy metabolism, based on patterns of expression and sequence analysis |
| Stb4p | Protein that binds Sin3p in a two-hybrid assay; contains a Zn(II)2Cys6 zinc finger domain characteristic of DNA-binding proteins; computational analysis suggests a role in regulation of expression of genes encoding transporters |
| Met28p | Basic leucine zipper (bZIP) transcriptional activator in the Cbf1p-Met4p-Met28p complex, participates in the regulation of sulfur metabolism |
| Gln3p | Transcriptional activator of genes regulated by nitrogen catabolite repression (NCR), localization and activity regulated by quality of nitrogen source |
| Ixr1p | Protein that binds DNA containing intrastrand cross-links formed by cisplatin, contains two HMG (high mobility group box) domains, which confer the ability to bend cisplatin-modified DNA; mediates aerobic transcriptional repression of COX5b |
| Gat1p | Transcriptional activator of genes involved in nitrogen catabolite repression; contains a GATA-1-type zinc finger DNA-binding motif; activity and localization regulated by nitrogen limitation and Ure2p |
| Abf1p | DNA binding protein with possible chromatin-reorganizing activity involved in transcriptional activation, gene silencing, and DNA replication and repair |
| Rsf2p | Zinc-finger protein involved in transcriptional control of both nuclear and mitochondrial genes, many of which specify products required for glycerol-based growth, respiration, and other functions |
| Pho4p | Basic helix-loop-helix (bHLH) transcription factor of the myc-family; activates transcription cooperatively with Pho2p in response to phosphate limitation; binding to 'CACGTG' motif is regulated by chromatin restriction, competitive binding of Cbf1p to the same DNA binding motif and cooperation with Pho2p; function is regulated by phosphorylation at multiple sites and by phosphate availability |
| Put3p | Transcriptional activator of proline utilization genes, constitutively binds PUT1 and PUT2 promoter sequences and undergoes a conformational change to form the active state; has a Zn(2)-Cys(6) binuclear cluster domain |
| Mot3p | Nuclear transcription factor with two Cys2-His2 zinc fingers; involved in repression of a subset of hypoxic genes by Rox1p, repression of several DAN/TIR genes during aerobic growth, and repression of ergosterol biosynthetic genes; can form the [MOT3+] prion |
| Gat4p | Protein containing GATA family zinc finger motifs |
| Cup9p | Homeodomain-containing transcriptional repressor of PTR2, which encodes a major peptide transporter; imported peptides activate ubiquitin-dependent proteolysis, resulting in degradation of Cup9p and de-repression of PTR2 transcription |
| Thi2p | Zinc finger protein of the Zn(II)2Cys6 type, probable transcriptional activator of thiamine biosynthetic genes |
| Reb1p | RNA polymerase I enhancer binding protein; DNA binding protein which binds to genes transcribed by both RNA polymerase I and RNA polymerase II; required for termination of RNA polymerase I transcription |
| Fkh2p | Forkhead family transcription factor with a major role in the expression of G2/M phase genes; positively regulates transcriptional elongation; negative role in chromatin silencing at HML and HMR; substrate of the Cdc28p/Clb5p kinase |
| Uga3p | Transcriptional activator necessary for gamma-aminobutyrate (GABA)-dependent induction of GABA genes (such as UGA1, UGA2, UGA4); zinc-finger transcription factor of the Zn(2)-Cys(6) binuclear cluster domain type; localized to the nucleus |
| Hcm1p | Forkhead transcription factor that drives S-phase specific expression of genes involved in chromosome segregation, spindle dynamics, and budding; suppressor of calmodulin mutants with specific SPB assembly defects; telomere maintenance role |
| Ngg1p | Transcriptional regulator involved in glucose repression of Gal4p-regulated genes; component of transcriptional adaptor and histone acetyltransferase complexes, the ADA complex, the SAGA complex, and the SLIK complex |
| Mot2p | Subunit of the CCR4-NOT complex, which has roles in transcription regulation, mRNA degradation, and post-transcriptional modifications; with Ubc4p, ubiquitinates nascent polypeptide-associated complex subunits and histone demethyase Jhd2p |
| Sok2p | Nuclear protein that plays a regulatory role in the cyclic AMP (cAMP)-dependent protein kinase (PKA) signal transduction pathway; negatively regulates pseudohyphal differentiation |
| Gcr1p | Transcriptional activator of genes involved in glycolysis; DNA-binding protein that interacts and functions with the transcriptional activator Gcr2p |
| Adr1p | Carbon source-responsive zinc-finger transcription factor, required for transcription of the glucose-repressed gene ADH2, of peroxisomal protein genes, and of genes required for ethanol, glycerol, and fatty acid utilization |
| Ime1p | Master regulator of meiosis that is active only during meiotic events, activates transcription of early meiotic genes through interaction with Ume6p, degraded by the 26S proteasome following phosphorylation by Ime2p |
| Msa2p | Putative transcriptional activator, that interacts with G1-specific transcription factor, MBF and G1-specific promoters; ortholog of Msa2p, an MBF and SBF activator that regulates G1-specific transcription and cell cycle initiation |

**Table S3** Primers used in this study

| primers | Sequences (5’-3’) |
| --- | --- |
| M13-47 | CGCCAGGGTTTTCCCAGTCACGAC |
| RV-M | GAGCGGATAACAATTTCACACAGG |
| K270R/N272D-F | GCCATCATTCCAAGGTCCGACACTGTCCC |
| K270R/N272D-R | GGGACAGTGTCGGACCTTGGAATGATGGC |
| XYL1t-prefix | AACTGCAGAACCATCACCTGCACAC**CAGT**CAGTTCGAGTTTATCATTATCA |
| XYL1t-suffix | GGAATTCCATATGGACCACCTGCGTAC**CATT**TCAATCAATGAATCGAAAATGTC |
| XYL2t-prefix | AACTGCAGAACCATCACCTGCACAC**AATG**CAGTTCGAGTTTATCATTATCA |
| XYL2t-suffix | GGAATTCCATATGGACCACCTGCGTAC**CTCA**TCAATCAATGAATCGAAAATGTC |
| XKS1t-Prefix | AACTGCAGAACCATCACCTGCACAC**TGAG**CAGTTCGAGTTTATCATTATCA |
| XKS1t-Suffix | GGAATTCCATATGGACCACCTGCGTAC**AAAA**TCAATCAATGAATCGAAAATGTC |
| LacZ-F | CCCAAGCTTGATAAATATCAAGCTCGAGC**CAGT**GTGTGCAGGTGAATTGTGAGCGGATAACAATTTCAC |
| LacZ-R | CGCGGATCCGCTAGATTTTGCATTGCTCCT**AAAA**GTACGCAGGTGGACAGTATCGGCCTCAGGAAGATC |
| pUC19-F | ATTGTCTCATGAGCGGATACATATTTG |
| pUC19-R | TCAGACCAAGTTTACTCATATATACTTTAGATTG |
| pUC19kan-F | GTATCCGCTCATGAGACAATAAGCCAGTTACCTTCGGAAAAAGAG |
| pUC19kan-R | TATGAGTAAACTTGGTCTGATTAATTCTTAGAAAAACTCATCGAGCATC |
| 6005 | GATCATTTATCTTTCACTGCGGAGAAG |
| 6006 | GTTTTAGAGCTAGAAATAGCAAGTTAAAATAAGGCTAGTC |
| URA-F | TATTCTTAACCCAACTGCACAGAACAAAAACCTGCAGGAAACGAAGATAAATCATGTCGAAAGCTACATATAAGGAACGGCTGCAGGTCGACAACCCTTAATATA |
| URA-R | TGAAGCTCTAATTTGTGAGTTTAGTATACATGCATTTACTTATAATACAGTTTTTTAGTTTTGCTGGCCGCATCCGACTCACTATAGGGCGAATT |
| PHO13U | GGTGAATGTTCTTTCCGTTTTAGTGAATTTTTCAATTGTAATTGACGCAATCGGTTTATAACAAGCAGACATAAATATCAAGCTCGAGCC |
| PHO13D | ATCAAATCATACAACTTACATAAAAACAACAAACCTGAATATTTTTCCTTTTCAAAAAGTAATTCTACCCCTAGATTTTGCATTGCTCCT |
| PHO13_tgR-F | TGCGCATGTTTCGGCGTTCGAAACTTCTCCGCAGTGAAAGATAAATGATC**TTCAACACCGAATTTCATAT**GTTTTAGAGCTAGAAATAGCAAGTTAAAATAAGGCTAGTCCGTTATCAAC |
| PHO13_tgR-R | GTTGATAACGGACTAGCCTTATTTTAACTTGCTATTTCTAGCTCTAAAAC**ATATGAAATTCGGTGTTGAA**GATCATTTATCTTTCACTGCGGAGAAGTTTCGAACGCCGAAACATGCGCA |
| XR-S | TATTGCTGTCACCGCTTAC |
| XR-A | GGAGACTTACCGTGCTTAG |
| XDH-S | GCTACTCCTAACTCCAAG |
| XDH-A | AGACAATGGCTCAACAAG |
| XK-S | AGCCGAATCTCTGTTAG |
| XK-A | GCACTCTTCAAACTCTTGAC |
| ENO1-S | GCTTTCGTTAAGGCTAACATTG |
| ENO1-A | AAGAAACACCCAAGATAGCG |
| ENO2-S | CGCTATCTTGGGTGTCTCCA |
| ENO2-A | GCACCAGTTGGAGCAATCAT |
| ACT1-S | ATGCAAACCGCTGCTCAA |
| ACT1-A | AGTTTGGTCAATACCGGCAGA |

The underlined bold 4 bases are overhangs compatible with the overhangs from the adjacent fragments or the acceptor vector; The bold 20 bases are target sequences of guide RNA targeting *PHO13* loci.


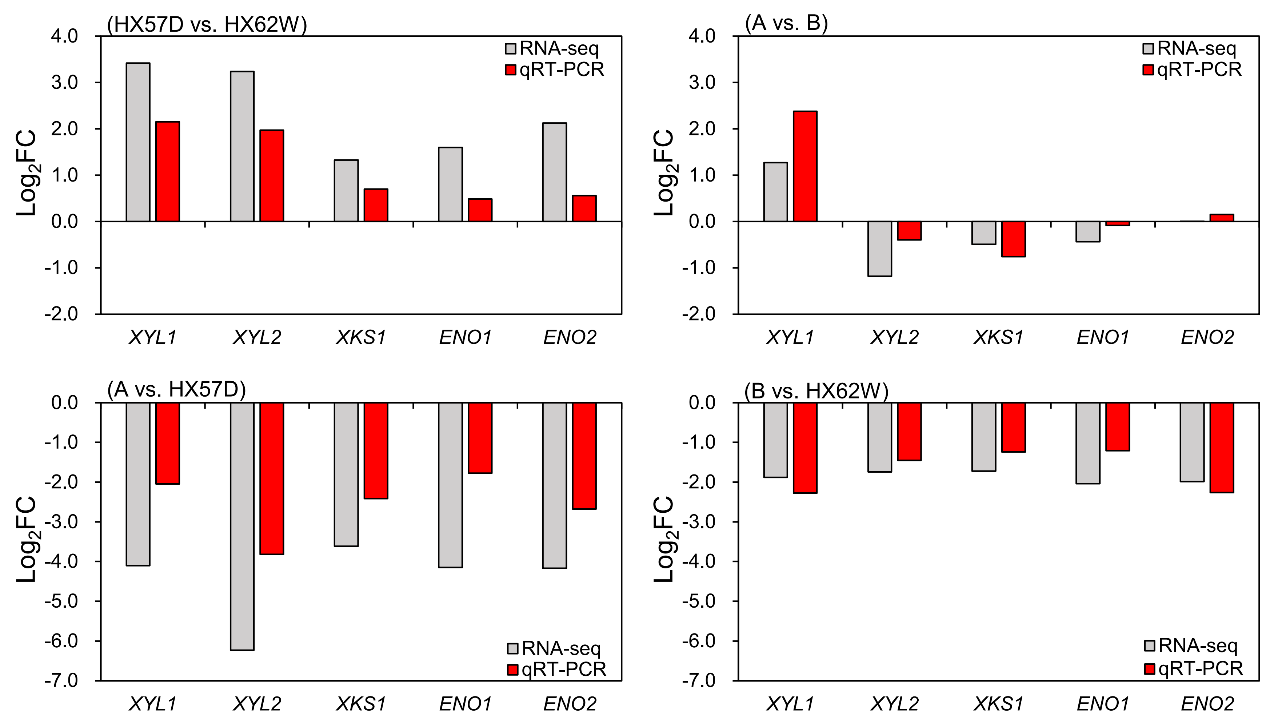


**Fig. S1** Validation of transcriptome data by real-time qRT-PCR. Fold change (FC) is the ratio of transcription level of specific gene in experimental group to that in control. *ACT1* was used as a reference gene.

**
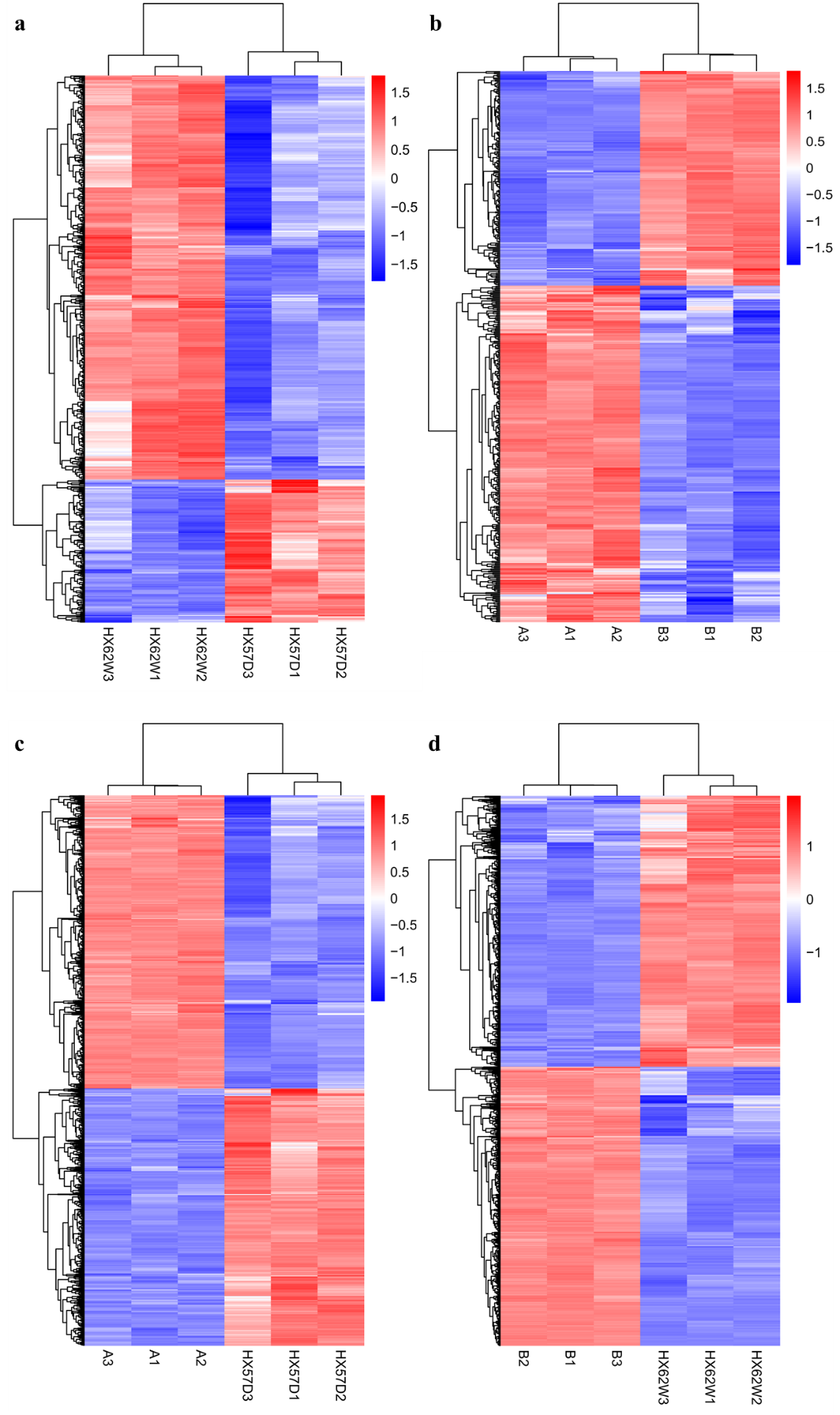
**

**Fig. S2** Cluster analysis of DEGs involved in comparison groups (**a**) HX57D vs. HX62W; (**b**) A vs. B; (**c**) A vs. HX57D; and (**d**) B vs. HX62W. Three biological replicates were carried out for each sample.
